# Establishment and genomic characterization of the new chordoma cell line Chor-IN-1

**DOI:** 10.1038/s41598-017-10044-3

**Published:** 2017-08-23

**Authors:** Roberta Bosotti, Paola Magnaghi, Sebastiano Di Bella, Liviana Cozzi, Carlo Cusi, Fabio Bozzi, Nicola Beltrami, Giovanni Carapezza, Dario Ballinari, Nadia Amboldi, Rosita Lupi, Alessio Somaschini, Laura Raddrizzani, Barbara Salom, Arturo Galvani, Silvia Stacchiotti, Elena Tamborini, Antonella Isacchi

**Affiliations:** 1 Oncology, Nerviano Medical Sciences, Nerviano, (MI) Italy; 2 Fondazione IRCCS Istituto Nazionale dei Tumori, Milan, Italy; 3 L.C. Laboratori Campisi, Avola (SR), Italy

## Abstract

Chordomas are rare, slowly growing tumors with high medical need, arising in the axial skeleton from notochord remnants. The transcription factor “brachyury” represents a distinctive molecular marker and a key oncogenic driver of chordomas. Tyrosine kinase receptors are also expressed, but so far kinase inhibitors have not shown clear clinical efficacy in chordoma patients. The need for effective therapies is extremely high, but the paucity of established chordoma cell lines has limited preclinical research. Here we describe the isolation of the new Chor-IN-1 cell line from a recurrent sacral chordoma and its characterization as compared to other chordoma cell lines. Chor-IN-1 displays genomic identity to the tumor of origin and has morphological features, growth characteristics and chromosomal abnormalities typical of chordoma, with expression of brachyury and other relevant biomarkers. Chor-IN-1 gene variants, copy number alterations and kinome gene expression were analyzed in comparison to other four chordoma cell lines, generating large scale DNA and mRNA genomic data that can be exploited for the identification of novel pharmacological targets and candidate predictive biomarkers of drug sensitivity in chordoma. The establishment of this new, well characterized chordoma cell line provides a useful tool for the identification of drugs active in chordoma.

## Introduction

Chordomas are malignant bone tumors that arise along any region of the axial skeleton, more frequently at sacro-coccygeal or skull-base ends^1, 2^. They are rare tumors, accounting for 1–4% of bone cancers, that occur more frequently in males than females and have a peak of incidence at around 60 years of age, although adolescents and children can also be affected^3^. Despite being slow progressing, chordomas are locally aggressive and invasive, and can also spread distally generating metastases in soft tissues, liver, lung, lymph-nodes and skin^1, 2^. No therapeutic options have so far revealed to be efficacious for this indication, which is highly resistant to conventional chemotherapy, and consequently no approved standard of care exists. Extensive surgery and/or high dose radiotherapy is currently used to treat the disease, but tumor location, frequently close to cranial or lombo-sacral nerves and blood vessels, makes the achievement of negative surgical margins very challenging^3–5^. In most cases local relapses occur and represent the main cause of death for patients^6, 7^. Chordomas therefore retain a strong unmet medical need, and new efficacious therapies are urgently needed.

Chordomas are thought to originate from remnants of the notochord, a transient structure required for patterning the surrounding tissues in all chordate embryos, that disappears later in development and is absent in adults. Among other developmental signals, notochordal cells express the transcription factor brachyury, a key molecule for mesoderm specification that is silenced in adult tissues^8^. Brachyury re-expression in notochord remnants is believed to play a major role in chordoma onset and maintenance and its expression is considered the main distinctive molecular marker of chordomas^9^. Brachyury expression is therefore considered a mandatory feature for the validation of chordoma cell lines^10^. Chordomas consistently also express other molecular markers, such as epithelial membrane antigen (EMA), vimentin, cytokeratin 19, CD24v and CAM5.2^3, 8, 11^. Moreover, variable expression and activation of several receptor tyrosine kinases (RTKs) and downstream signaling molecules have been reported. Among these, EGFR, PDGFR and MET represent the most frequently expressed and activated RTKs in chordoma^12–14^.

Very few bona fide chordoma cell lines have been available until recently, limiting the identification of relevant targets and the development of effective drugs. Validated chordoma cell lines available from the Chordoma Foundation (a global chordoma patient advocacy group, www.chordomafoundation.org) include the prototype cell lines U-CH1 and U-CH2 and a few other more recently established cell lines^15–17^.

Here we describe the generation and characterization of the new Chor-IN-1 chordoma cell line, established from a surgical sample of a sacral chordoma. The Chor-IN-1 cell line was shown to display the morphological and growth features of chordoma and to express brachyury, as well as other key relevant markers associated with chordoma diagnosis. This newly established cell line was characterized in parallel with U-CH1^15^, U-CH2^15^, MUG-Chor1^16^ and JHC7^17^ chordoma cell lines by Next Generation Sequencing (NGS), in order to compare genomic profiles and to evaluate the expression of kinases which might represent potential new therapeutic targets.

## Results

### Chor-IN-1 cell line establishment

**Figure 1:**
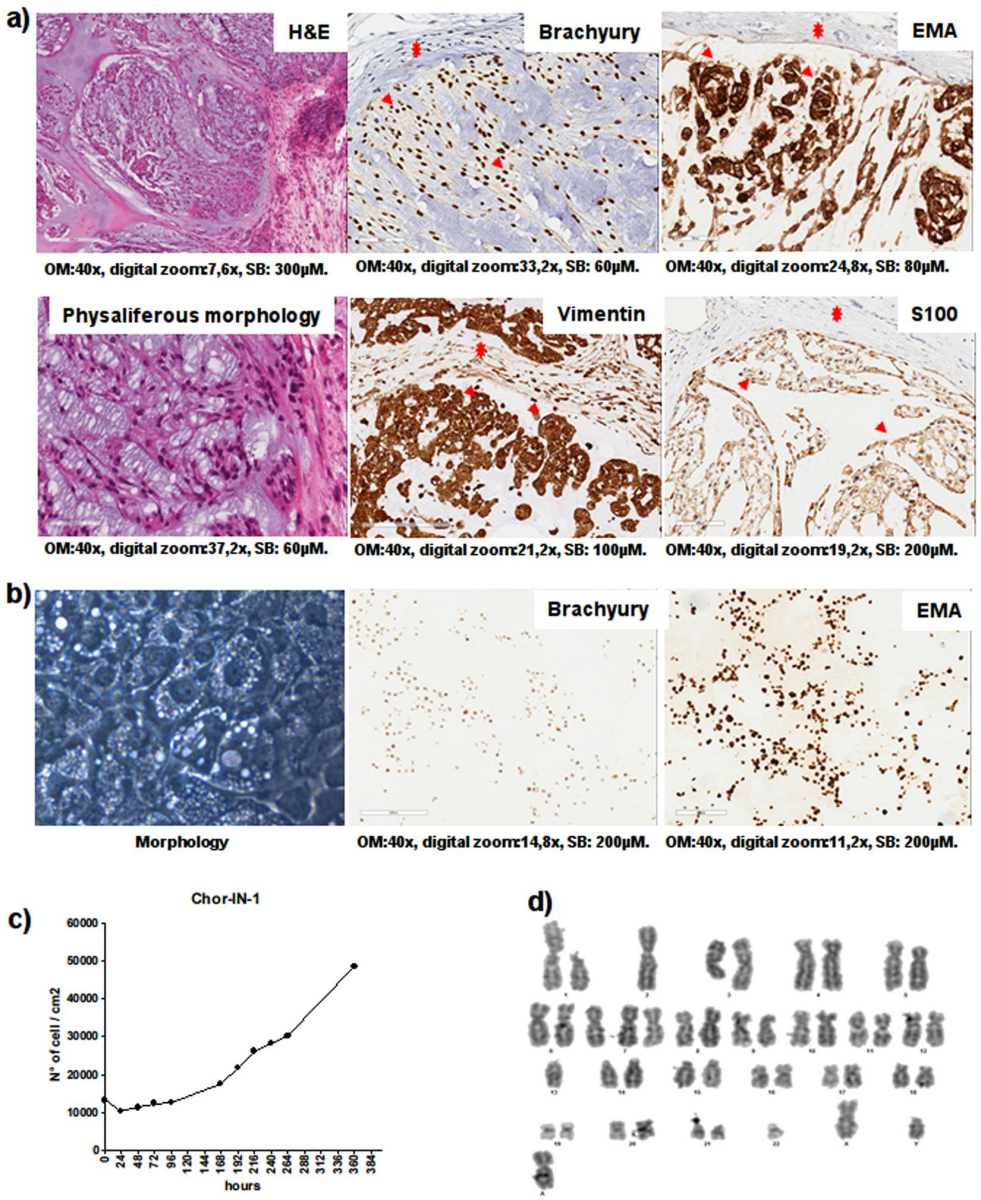
Tumor and Chor-IN-1 cell line characterization by H&E and IHC. (**a**) The original tumor sample and (**b**) the derived Chor-IN-1 cell line were characterized by H&E, revealing the typical physaliferous cells, and by IHC, showing positivity for brachyury and for other chordoma typical biomarkers, as indicated. In (**a**), arrows and stars indicate examples of tumor and fibroblast cells, respectively. (**c**) Growth curve: the doubling time of Chor-IN-1 cell line was calculated as reported and found to be of about seven days. (**d**) Karyotype analysis of Chor-IN-1 cell line.

The original surgical sample was obtained from a 55 year old man diagnosed with a locally advanced sacral chordoma. A sacral nodule of 2 cm of diameter, macroscopically invading the surrounding soft tissues, was surgically excised. Histological diagnosis of chordoma was made according to WHO classification (2013). Morphologically, the tumor recapitulated the features of conventional chordoma, exhibiting lobulated growth of round epithelioid cells separated by fibrous septa. The cells, arranged in ribbons and nests, showed eosinophilic and/or vacuolated cytoplasms (physaliferous morphology) and were embedded into abundant extracellular matrix. Immuno-phenotyping revealed expression of vimentin, S100, brachyury and EMA (Fig. 1a).

The new cell line was established by mechanical and enzymatic disaggregation of the fresh aseptic surgical chordoma sample followed by seeding of the resulting cell suspension in collagen-coated tissue culture plates. Once stabilized in culture, the Chor-IN-1 cell line was subjected to detailed characterization. The morphology was repeatedly monitored over passages, confirming that most of the cells displayed the typical physaliferous phenotype (Fig. 1b) of chordoma cells. This physaliferous morphology was less evident immediately after seeding but involved most of the cells 2–3 days later and is likely associated with non-actively dividing cells, as previously reported for the MUG-Chor1 cell line^18^. The cell line was confirmed to express brachyury and EMA by immunocytochemistry (Fig. 1b).

The Chor-IN-1 cell line displayed a doubling time of about seven days (Fig. 1c), in agreement with the very slow growth rate typical of chordoma cell lines that faithfully represent the disease^15^.

The karyotype of the cell line was analyzed using Cytovision® software and found to be: 45, XY, add(1)(p13), −2, add(3)(q21), +7, del(9)(p21), −13, −22, +mar[cp10], a pseudo-diploid karyotype with chromosomal numerical and structural abnormalities typically found in this tumor (namely: del 1p, monosomy 2, deletion 3q, trisomy 7, monosomy 13 and 22) (Fig. 1d)^9^.

### Chor-IN-1 cell line validation and molecular characterization

**Figure 2:**
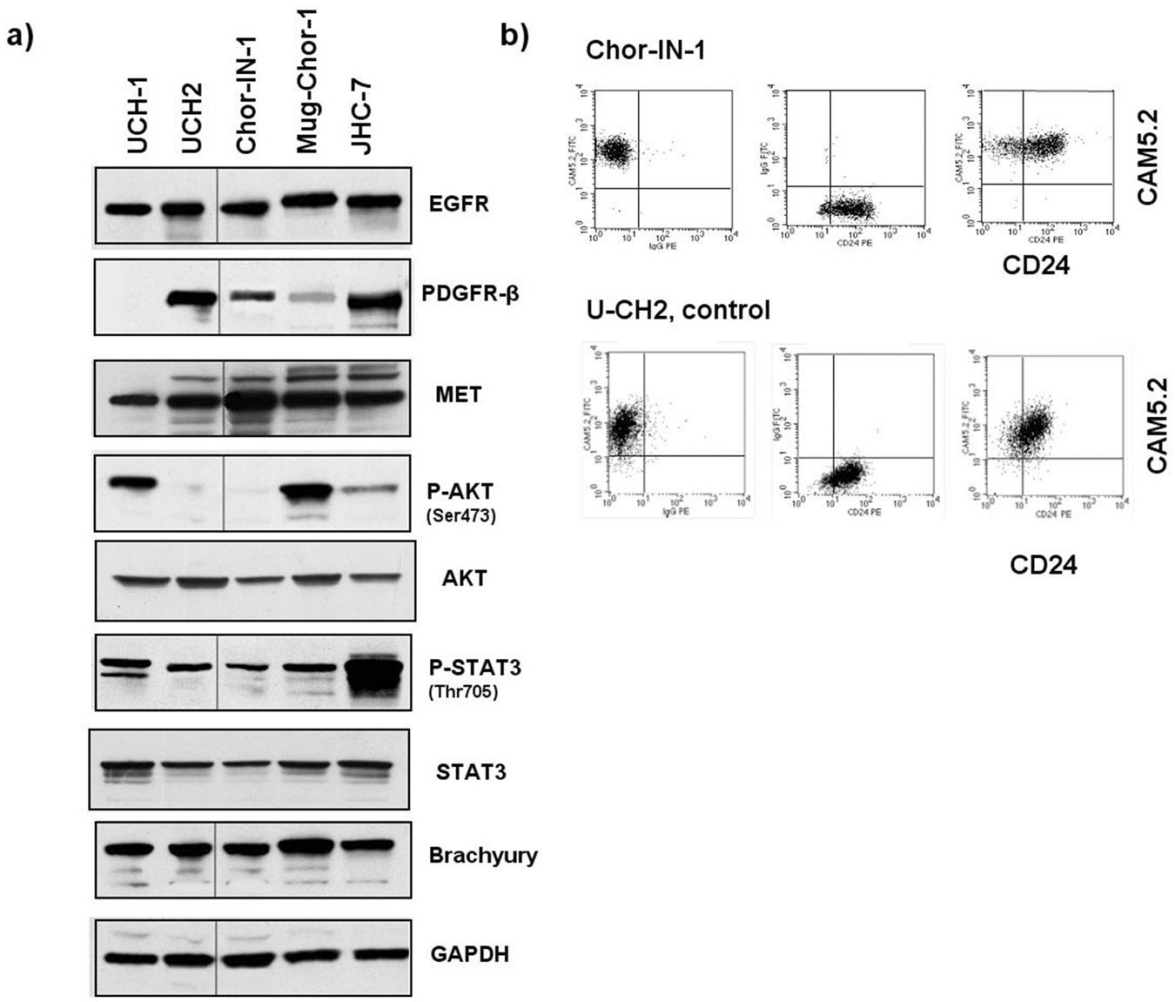
Molecular Characterization of Chor-IN-1 cell line. (**a**) Immunoblot analysis of key tyrosine kinase receptors and other signaling molecules: Chor-IN-1, in parallel with U-CH1, U-CH2, MUG-Chor1 and JHC7 cells were seeded as indicated and collected at 70% confluence. Protein cell extracts were resolved by SDS-PAGE and filters probed with the indicated antibodies. Full-length blots are presented in Supplementary Figure 6. (**b**) Flow Cytometry analysis of chordoma typical membrane proteins: the expression of CD24 and CAM5.2 membrane antigens in Chor-IN-1 was confirmed to be comparable to that of the U-CH2 cell line used as reference. Upper cytograms refer to Chor-IN-1 cells and lower cytograms refer to UCH-2 cells. Left = FITC CAM5.2 *vs*. PE isotype ctrl: 100% of both cell lines express the marker, in the absence of non-specific signals. Middle = PE-CD24 *vs*. FITC isotype ctrl: more than 90% of both cell lines express the marker, in the absence of non-specific signals. Right = FITC CAM5.2 *vs*. PE-CD24: both cell lines are more than 90% CD24/CAM5.2 double positive, confirming both markers are expressed at levels comparable to that of UCH-2 reference cell line.

The Chor-IN-1 cell line was then characterized for the expression of key RTKs and downstream signaling molecules, in comparison to the other chordoma cell lines U-CH1, U-CH2, MUG-Chor1 and JHC7. Western Blot analysis revealed that Chor-IN-1 expresses significant levels of EGFR, PDGFRβ and c-Met proteins, with activation of STAT3 and a very weak P-AKT (Fig. 2a).

Brachyury protein was expressed at comparable levels in all cell lines (Fig. 2a). Interestingly, RT-qPCR analysis revealed that Chor-IN-1 expresses mRNA levels of the T gene, encoding brachyury, comparable to U-CH1 and U-CH2. Conversely, the MUG-Chor1 and JHC7 cell lines showed higher mRNA expression levels which do not however translate into higher protein levels, likely indicating a strict cellular control on the protein levels of this transcription factor (Suppl. Fig. 1). Cytokeratin 19 and vimentin were also confirmed to be expressed by RT-qPCR analysis, as required for comprehensive chordoma cell line validation (Suppl. Fig. 1). Finally, FACS analysis confirmed the expression of CD24 and CAM5.2 membrane antigens in the Chor-IN-1 cell line, similar to the U-CH2 used as control cell line (Fig. 2b).

The Chor-IN-1 cell line was authenticated by Short Tandem Repeat (STR) analysis in parallel with the parental tumor sample. The identity of the other chordoma cell lines was also confirmed by comparison with the published STR profiles (Suppl. Fig. 2).

We were primarily interested in the identification of gene variants of clinical relevance, thus we used the Illumina TruSight One “clinical exome” panel (Illumina, San Diego, CA, USA), which allows the complete sequencing of the exonic regions of a subset of 4,813 genes harbouring disease-causing mutations, covering genes reported in the Human Gene Mutation Database (HGMD), in the Online Mendelian Inheritance in Man (OMIM) catalog and most of the genes currently reviewed in clinical research. Variants (SNVs and short InDels) detected in the Chor-IN-1 cell line and in the original tumor were highly consistent (>80%). All but four of these variants were also present in the DNA extracted from the non-tumor tissue component, therefore indicating that they represent germline variants. Tumor unique variants were identified in the MUC1, KEL, TECTA and SART3 genes (Suppl. Fig. 3). We also manually inspected the raw data for the 9 variants found uniquely in the normal sample. These variants could not be detected in the tumor sample and its derived cell line with the imposed coverage threshold, however they were called just below the filter threshold, due to their location in regions of lower coverage.

**Figure 3:**
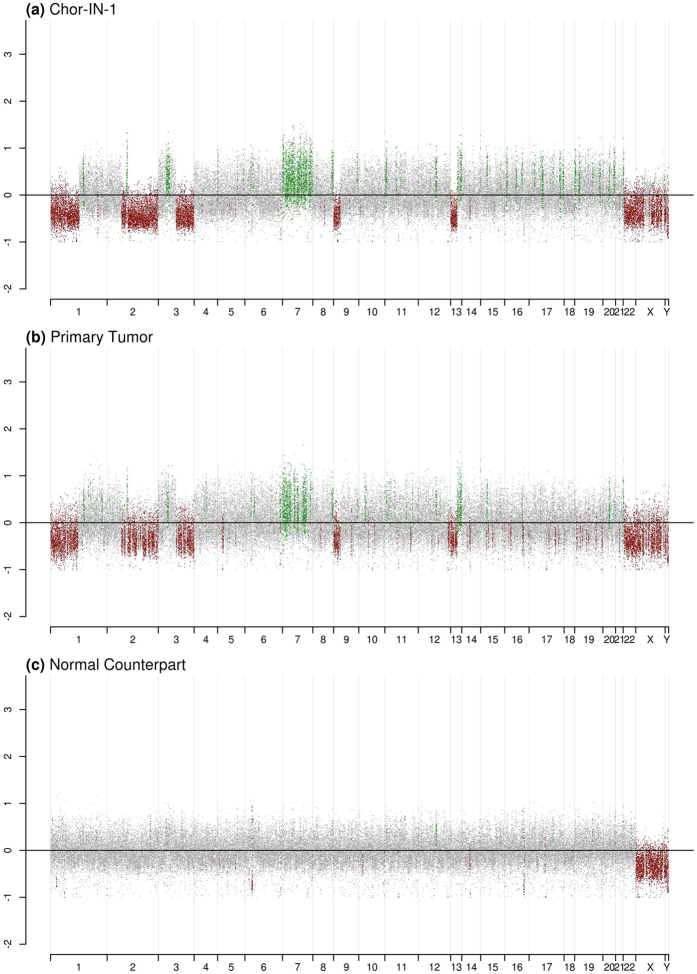
Genomic profile of Chor-IN-1 cell line and original tumor.Copy number profiles obtained by NGS analysis on: (**a**) the newly established Chor-IN-1 cell line, (**b**) the primary tumor of origin, (**c**) the normal tissue counterpart. Log2 ratio of the coverage in each interval *vs* medium coverage is reported in the plot. Regions with chromosomal gain (log2 ratio > 0.2) are reported in green, while regions of chromosomal loss (log2 ratio < −0.2) are reported in red. Balanced genomic regions are reported in grey.

Also, the overall Chor-IN-1 genomic profile was compared to the original tumor, revealing a complete overlap, with several major genomic alterations that were not present in the non-tumor tissue component (Fig. 3).

In summary, this extensive molecular characterization confirms that the Chor-IN-1 cell line faithfully recapitulates the molecular features of the tumor of origin.

In order to highlight similarities and differences to other chordoma lines, the Chor-IN-1 variants were compared to those identified by TruSight One sequencing performed on the other four cell lines (Suppl. Fig. 4). Only a few variants common to all chordoma cell lines were identified. However they could not be considered peculiar to chordoma, since they were also present in other unrelated tumor cell lines. Also, the four Chor-IN-1 tumor-specific variants were not present in the other chordoma cell lines. Therefore, we were unable to identify somatic mutations distinctive of chordoma, in line with what has already been published in literature^19, 20^.

NGS analysis of the Brachyury gene revealed the presence of a homozygous polymorphism (c.G530A) in the Chor-IN-1 and in all the other chordoma cell lines, which was also present in the original tumor tissue and in its normal component, as confirmed by Sanger sequencing (Suppl. Fig. 5). This polymorphism, resulting in a p.G177D amino acid substitution within the DNA binding domain, was previously reported to be associated to chordoma predisposition^21^.

### Copy number profiling of Chor-IN-1

**Figure 4:**
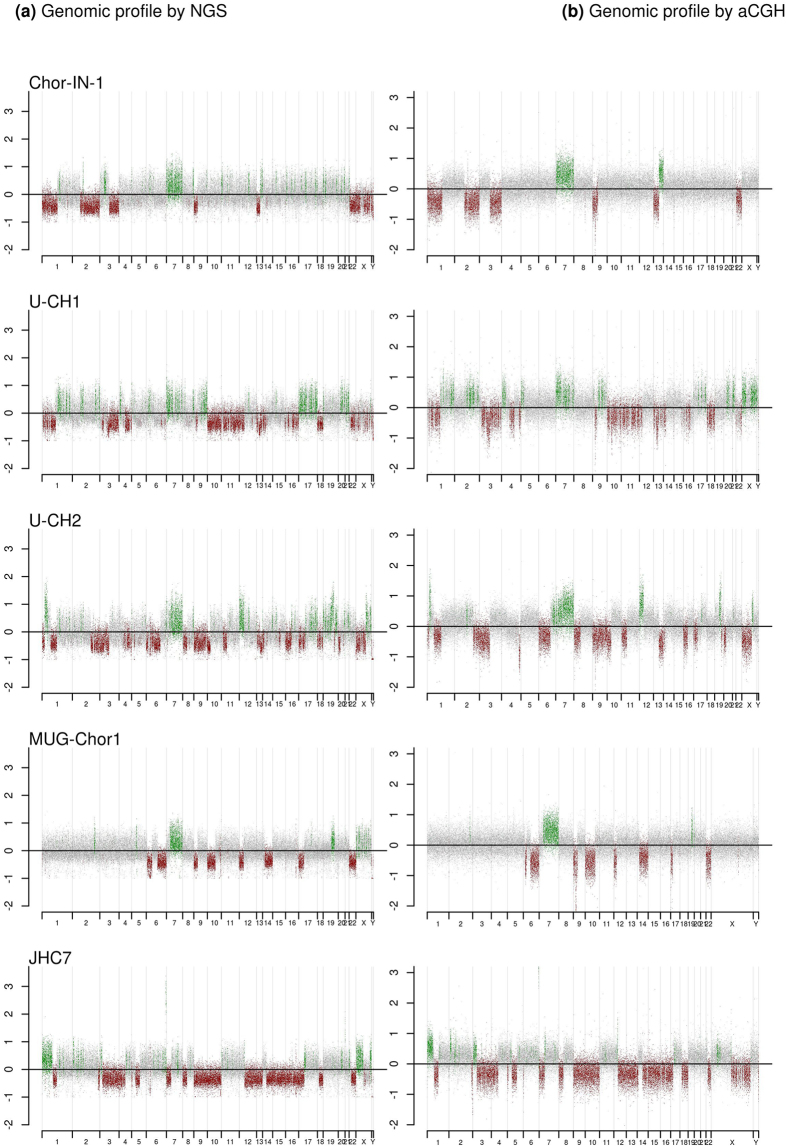
NGS and aCGH copy number profiles on the different chordoma cell lines.Copy number profiles of Chor-IN-1 cell line *vs*. the other chordoma cell lines obtained by NGS and aCGH analysis: (**a**) NGS analysis: the different cell lines were profiled by NGS using Illumina TruSight One “clinical exome” panel (Illumina, San Diego, CA, USA). Log2 ratio of the coverage calculated in each interval *vs*. medium coverage is reported in the plot. (**b**) aCGH analysis: the different cell lines were profiled by aCGH. The Array Comparative Genomic Hybridization (aCGH) characterization was performed using the oligonucleotide-based microarray CytoChip Cancer 8 × 60 kv2.0 for Chor-IN-1, U-CH1 and U-CH2 samples and CytoChip ISCA 8 × 60 K v2.0 for MUG-Chor1 and JHC7 samples (BlueGenome Ltd, Cambridge, UK). Log2 ratio of cell line (ch1) *vs*. reference (ch2) value was calculated. Regions with chromosomal gain (log2 ratio > 0.2) are reported in green, while regions of chromosomal loss (log2 ratio < −0.2) are reported in red. Balanced genomic regions are reported in grey.

The chromosomal rearrangements of the Chor-IN-1 cell line were then compared to those present in the other cell lines by NGS as well as by aCGH analysis. Results obtained with the two methods were superimposable (Fig. 4a,b). Regions of abnormality specific for the Chor-IN-1 cell line, as well as copy number aberrations common to other chordoma cell lines, were identified. In particular, Chor-IN-1 specific alterations were: (i) a monosomy of chr2q; (ii) a monosomy of chr3q; (iii) a microdeletion in chr8q21.3; (iv) a single locus amplification in chr11q13.1; and (v) a duplication in chr13q21.31-qter.

Chromosomal alterations shared by all cell lines but JHC7 included the monosomy of chr1p, the trisomy of chr7 and a biallelic deletion of 9p21. A monosomy of chr 22 could be observed in Chor-IN-1, U-CH1 and MUG-Chor1 cell lines. A monosomy of chr2q was observed in the Chor-IN-1 cell line, while a partial terminal deletion of chr2q was present in U-CH2 and JHC7. Also, a gain of the whole chromosome X was present in U-CH1, while a partial duplication of Xq and a monosomy of Xpter-q21.33 were detectable in U-CH2. Furthermore, only Chor-IN-1 and U-CH1 cell lines shared a monosomy of chr1p and a partial deletion of a chr13q region. A duplication of the 6q22.1-qter region could be appreciated in U-CH2. A gain of 6q27 region, including the brachyury gene, could be clearly detected in JHC7 cell line and to a lesser extent in MUG-Chor1, where a complex rearrangement including the amplification of the T locus is present, likely accounting for the higher levels of T gene mRNA in these two cell lines (Fig. 4 and Suppl. Fig. 1).

**Figure 5:**
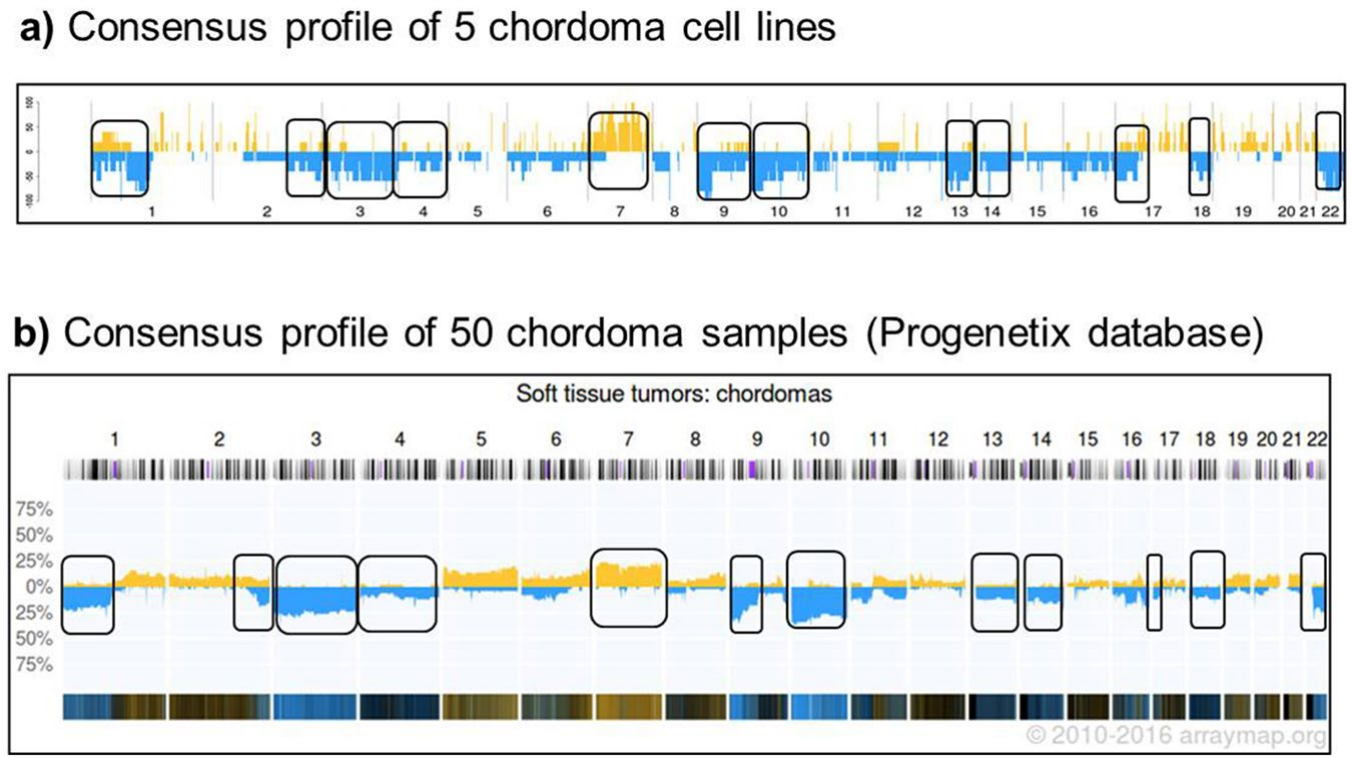
Comparison of chordoma cell lines *vs*. chordoma samples consensus profile.Comparison of the consensus profile obtained from all chordoma cell lines and the profile of the chordoma samples included in Progenetix database. (**a**) Consensus profile calculated using TSO coverage data from all the analyzed chordoma cell lines. Percentage of chromosomal aberration (gain or loss) obtained for each genomic interval in chordoma cell lines is reported on the y-axis of the plot. Regions of chromosomal gain are reported in yellow, while regions of chromosomal loss in blue. (**b**) Consensus profile of 50 chordoma clinical samples as obtained from Progenetix database.

The genomic similarity between chordoma cell lines and clinical samples of chordoma was then investigated by calculating a consensus profile of the chordoma cell lines based on the NGS data and comparing it to the consensus profile calculated by Progenetix on 50 chordoma tumor samples profiled by aCGH^22^; www.progenetix.org (Fig. 5). Consensus copy number aberrations observed in the cell lines were generally consistent with those identified in the clinical tumor samples, including deletions of regions within chromosomes 1, 2, 3, 4, 9, 10, 13, 14, 17, 18 and 22 and a major amplification in chromosome 7, indicating that the cell line panel used in this study recapitulates the major chordoma tumor features.

### Kinase gene expression analysis of Chor-IN-1

The detection of abundantly/differentially expressed kinases in the chordoma cell lines might allow the identification of potential new pharmacological targets in this disease. For this reason, we analyzed the global kinase gene expression of Chor-IN-1 in parallel with the other cell lines applying a custom targeted RNA sequencing approach (TREx, Illumina, San Diego, CA, USA) to investigate the kinome gene expression in chordoma (~ 500 kinases). Gene expression values for the whole kinome are provided as Supplementary Table 1.

**Figure 6:**
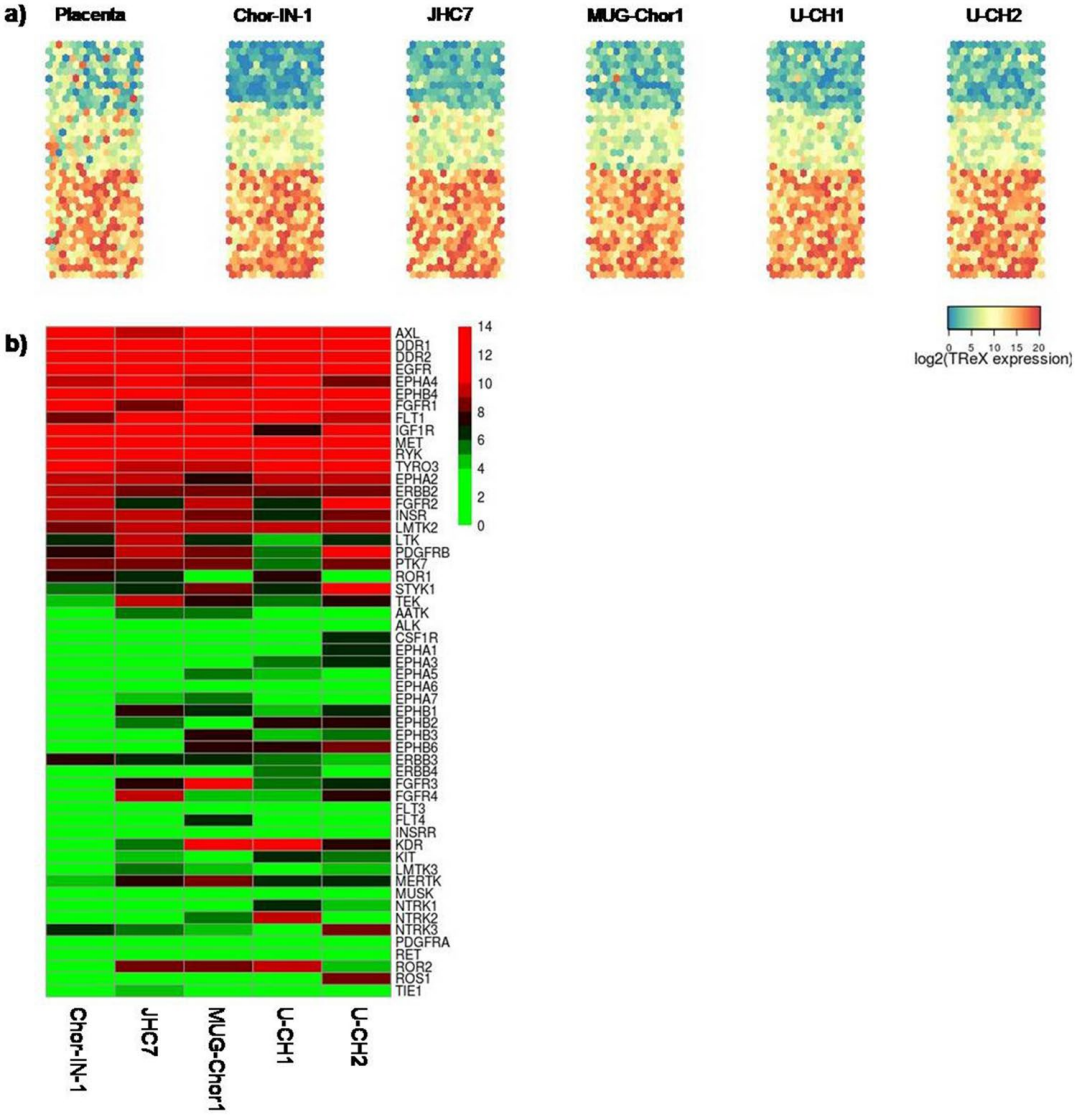
Kinase gene expression levels in chordoma cell lines. (**a**) Kohonen maps reporting gene expression levels of 487 kinases in the different chordoma cell lines. Placenta was used as control sample. Each dot represents an individual kinase whose position is kept constant in the map. Colormap gradient from blue to red reflects increasing gene expression level, expressed in log2 scale. (**b**) Heatmap reporting gene expression levels of RTKs (log2 scale) sorted from high to low levels. Colours reflect gene expression levels according to reported scale.

Results were displayed using Kohonen maps, a data analysis and visualization neural network- based technique for multidimensional quantitative and qualitative data comparison (https://cran.r-project.org/web/packages/kohonen/index.html). This analysis showed a very consistent kinase expression profile among chordoma cell lines, which differs from that of control placenta tissue (Fig. 6a). As shown in Fig. 6a, in the chordoma panel, about 75% of kinases are expressed, more than half at high levels, while 25% are expressed at very low levels or not expressed.

**Figure 7:**
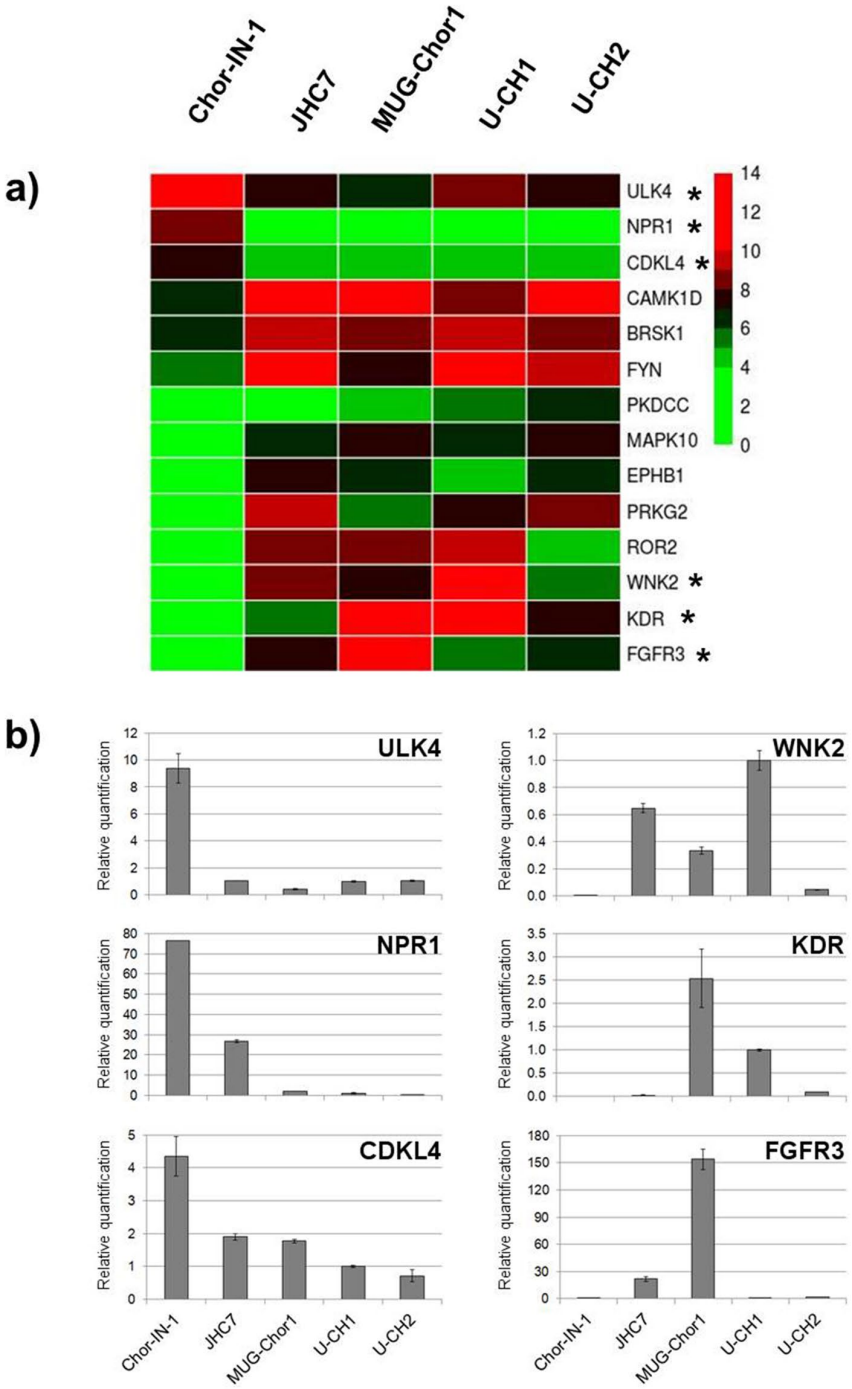
Identification of kinases differentially expressed in Chor-IN-1 cell line *vs*. a panel of chordoma cell lines. (**a**) Heatmap representing the expression levels of the most differentially expressed kinases in Chor-IN-1 cell line, as obtained by NGS analysis using TREx, Illumina, San Diego, CA, USA. Colormap shades reflect relative low to high expression levels, ranging from green (log2 = 0) to red (log2 = 14). (**b**) RT-qPCR validation of selected genes (indicated with a star in the heatmap). Histogram bars represent RT-qPCR relative quantification results, carried out as described in M&M and normalized using U-CH1 as a reference sample.

We focused our analysis particularly on RTKs, which are involved in cell regulation processes and frequently dysregulated in tumors. We found that out of 56 RTKs, 15 were expressed at high levels in all cell lines. These include EGFR and MET, as expected, while PDGFR-β was expressed in all cell lines but not in the U-CH1 (Fig. 6b). A few kinases showed a cell-line distinctive profile. Therefore we further analyzed the kinases specifically expressed in the newly established Chor-IN-1 cell line by gene expression differential analysis. The heatmap depicted in Fig. 7a shows the differentially expressed kinases in Chor-IN-1 *vs*. all the other chordoma cell lines (pV < 0.05, FC > |2|). ULK4, NPR1 and CDKL4 were the top most expressed kinases in the Chor-IN-1 cell line, while FGFR3, KDR and WNK2 were less expressed or absent in Chor-IN-1 cells as compared to the other chordoma lines. The differential expression of these kinases in Chor-IN-1 was confirmed by RT-qPCR (Fig. 7b).

## Discussion

Chordoma is a rare disease with a high unmet medical need. Preclinical research on chordoma has been hampered by the limited number of available cell lines, which were only recently expanded beyond the two prototype U-CH1 and U-CH2 cell lines. The availability of well characterized, newly-established cell lines can have a high impact on understanding the complex biology of this tumor, potentially contributing to the identification of new therapeutic targets.

Here we present the establishment of a novel chordoma cell line from a sacral tumor sample, which was named Chor-IN-1. We also provide an extensive molecular characterization of this cell line in comparison to the original tumor and to a panel of representative chordoma cell lines.

In keeping with the indolent nature of this tumor, chordoma cell lines display very long doubling times in culture and require several months to reach stabilization. The Chor-IN-1 cell line has been maintained *in vitro* for >50 population doublings. This is a required feature for consideration as a stabilized chordoma cell line, since chordoma primary cell cultures tend to enter a growth crisis after 25–30 passages *in vitro* (www.chordomafoundation.org).

We decided to characterize Chor-IN-1 in parallel with four other cell lines of sacral origin, including the most widely used U-CH1 and U-CH2, as well as the more recently established MUG-Chor1 and JHC7 cell lines.

In general, the TruSight One analysis did not identify gene variants common to the different cell lines, in agreement with literature data^19^. Also, the availability of patient DNA from the non tumoral tissue highlighted that the SNVs identified in the tumor tissue and Chor-IN-1 cell line were mainly germline, with four exceptions involving TECTA, SART3, KEL and MUC1 genes. Interestingly, MUC1 (Mucin 1, Cell Surface Associated) is the gene encoding EMA, a transmembrane glycoprotein, commonly used as a biomarker for chordoma diagnosis^17, 23^. This variant lies within an insertion region of unknown significance, located within the signal peptide present in a subset of MUC1 isoforms. The biological significance of the mutations identified in these genes will require further studies.

Next we investigated gross chromosomal rearrangements by mean of aCGH and NGS analysis. Several chromosomal alterations shared among the chordoma cell lines were identified. In particular, all chordoma cell lines apart from JHC7 share a biallelic loss of 9p21. This region includes the loci for CDKN2A and CDKN2B tumor suppressor genes and is reported to be frequently altered in chordoma tumor samples (~59%)^24^.

Similarly, trisomy of chr7, clearly evidenced also by karyotype analysis of the Chor-IN-1, is a feature shared by all the chordoma cell lines with the exception of JHC7, which bears a partial chr7 trisomy involving only the long arm. This trisomy was previously reported in a significant fraction of chordoma clinical samples^25, 26^. Interestingly, the EGFR gene is located in the short arm of chr7 and is therefore not affected by the partial trisomy in the JHC7 cell line. In general, the major chromosomal abnormalities, common to the chordoma cell lines reported here, well recapitulate the most common alterations reported in Progenetix database for chordoma clinical samples.

Besides these features, typical of chordoma, the Chor-IN-1 genome harbors peculiar chromosomal alterations in regions containing bone dysmorphism-associated genes, including DACH1, a gene involved in regulation of gene expression and cell fate determination during development, reported to downregulate EGFR and cyclin D1 and associated with osteosarcoma development^27^; LIG4, whose mutations are responsible for LIG4 syndrome, a disease characterized by dysmorphic features and microcephaly^28^ and WWP1, an E3 ubiquitin ligase involved in the regulation of osteoblast functions^29^. Of interest, an evident amplification of the MALAT1 locus, associated with the proliferation and metastasis of tumor cells, was observed.

Chor-IN-1 major alterations were also detected by karyotype analysis. In particular, this analysis revealed the presence of an anomalous submetacentric C-like chromosome (chromosome A), possibly originated from the rearrangement of different portions of other chromosomes. This might account for the presence of the short arm of chr2 and of partial long arm of chr13 observed in aCGH analysis and not in the karyotype. Interestingly, chordomas were described as being among tumors that undergo “chromothripsis”, a peculiar event involving massive genomic rearrangements of one or few chromosomes^30^. It is tempting to speculate that the newly identified “chromosome A” might have been assembled from portions of different chromosomes following a catastrophic chromothriptic event that occurred in the development of this tumor.

We next focused on the characterization of protein kinase gene expression in chordoma cell lines. Kinases constitute one of the largest known families of enzymes that control different cellular functions and whose deregulation plays a causal role in cancer. A number of receptor tyrosine kinases have been reported to be implicated in chordoma pathogenesis, including EGFR, PDGFRβ, and c-MET^13, 14, 31^. We reasoned that detection of abundantly/differentially expressed kinases in chordoma cell lines might represent a convenient strategy for the identification of potential new pharmacological targets in this disease.

We provide here data on kinome gene expression in the different chordoma cell lines, which can be exploited to investigate the molecular basis for the sensitivity of each cell line to the different kinase inhibitors. Several among the most widely expressed kinases are inhibited by drugs currently undergoing clinical development, which may represent a new therapeutic option also in chordoma. We recently investigated in depth the role of EGFR inhibitors, providing a rationale to start a clinical trial with afatinib in this setting (Magnaghi P. *et al*., manuscript submitted). The current analysis highlighted AXL, DDR1, DDR2, EPHA4, EPHB4, EPHA2, FGFR1, FGFR2, MET and ERBB2 as other interesting kinases that deserve further investigation as potential biologically relevant targets in chordoma.

Comparative analysis focused on Chor-IN-1 showed that this cell line expresses high levels of ULK4, a member of the unc-51-like serine/threonine kinase (STK) family. Although little is known about ULK4 function, its role in chordoma deserves further investigation since the other members of the family have been implicated in autophagic pathways. CDKL4 (Cyclin Dependent Kinase Like 4), a member of the CDK family which includes CDK4 and CDK6, is also highly expressed. Interestingly, the use of CDK4/6-specific inhibitor palbociclib was reported to efficiently inhibit tumor cell growth *in vitro* in chordoma cell line models^32^, providing the rationale for clinical trials evaluating the efficacy of palbociclib in chordoma.

In conclusion, we generated and extensively characterized Chor-IN-1, a new chordoma cell line that represents a valuable contribution to the preclinical research in the field, in view of the paucity of current cell models and of the heterogeneity of chordoma tumors. The Chor-IN-1 cell line will be made available to be used for preclinical studies aimed at further understanding the pathogenesis of chordoma and its sensitivity to available drugs. Moreover, we have generated genomic data at the DNA and RNA level that can be exploited for the identification of biomarkers of drug sensitivity, and for the identification of novel pharmacological targets in chordoma.

## Methods

### Case report

The surgical sample was obtained from a patient initially diagnosed with sacral chordoma. The patient refused surgery, and received imatinib and radiotherapy. After three years, the patient experienced local subcutaneous progression at lombo-sacral level, received again imatinib and, upon further progression, also metformin. A tumor biopsy was performed when the patient finally underwent surgery. A sacral nodule of 2 cm of diameter, invading macroscopically the surrounding soft tissues, was surgically excised. All methods were performed in accordance with the relevant guidelines and regulations.

The patient gave his informed consent to study his tumor, followed by the approval of the Fondazione IRCCS Istituto Nazionale dei Tumori (Milan, Italy) Ethical Committee for the genotypic and phenotypic characterization of the cell line to confirm the identity with original tumor.

### Cell culture

Fresh aseptic surgical chordoma sample was minced and incubated with collagenase (Cat. No. C6885, Sigma) for three hours and the obtained cell suspension was filtered through a 45 µL nylon mesh, washed with PBS/FCS 0.5% and seeded on collagen coated plates using 70% DMEM 30% IMDM2 supplemented with 10% of fetal bovine serum. When confluence was reached, cells were carefully detached and passed in new flasks at 20,000 cells/cm^2^. Cells were monitored daily to evaluate growth and morphology, and were carefully detached and re-seeded upon reaching confluence. After three months in culture the resulting cell line, named Chor-IN-1, displayed stable morphological and growth features and was further maintained in culture up to 50 population doublings, which required fourteen months. For doubling time calculation, Chor-IN-1 cells were seeded into 12 well plates (13,000 cells/cm^2^) into 1 ml of culture medium. Cells were carefully detached from two wells every day and counted using a Multisize 3 (Beckman Coulter). Doubling time was calculated on the exponential part of the growth curve, using the formula DT = (Log_2_ Cell number[Tx] − Log_2_ Cell number[T0])/Tx (hr).

The other chordoma cell lines were obtained from the Chordoma Foundation (U-CH1, U-CH2, JHC7) or purchased from ATCC (MUG-Chor1). Chordoma cell lines were maintained using collagen-coated flasks (Cellcoat Cat 658950 Collagen Type I – Greiner Bio-one) in an appropriate culture medium (IMDM/RPMI 1640 4:1 ratio), supplemented with 10% (v/v) heat-inactivated fetal bovine serum (Mediatech, Inc. Corning – US - Lot 35015109) at 37 °C in a humidified atmosphere containing 5% CO_2_. Cultures were all routinely checked for mycoplasma contamination using MycoAlert (Lonza– USA).

### Reagents and Antibodies

The following antibodies were used: Anti-EGFR (Santa Cruz sc-03 rabbit polyclonal); anti-PDGFRβ (Cell Signaling 4564 rabbit monoclonal); anti-MET (Santa Cruz sc10 rabbit polyclonal); anti-P-AKT (Cell Signaling 4060 rabbit monoclonal); anti- AKT (Cell Signaling – cs 9272); anti-P-STAT3 (Cell Signaling 9131 rabbit polyclonal); anti-STAT3 (Cell Signaling – cs 9139); anti-brachyury (Santa Cruz sc-20109 rabbit polyclonal), anti- GAPDH (Santa Cruz 25778 rabbit polyclonal).

HRP-conjugated secondary antibodies were used 1:10000 (Immunopure goat anti-mouse and Immunopure Goat Anti-rabbit from Thermo-Scientific). Detection was performed using SuperSignal west Pico Chemiluminescent substrate from Thermo Scientific

### Immunohistochemistry (IHC)

Representative sections obtained from the formalin fixed paraffin embedded (FFPE) tumoral sample of the case were selected and phenotyped. IHC was done with 2-µm FFPE sections. Anti-Brachyury (cat. sc-20109, polyclonal antibody, Santa Cruz) and Anti-vimentin (Clone V9, DAKO), were diluted 1:400, while Anti-EMA (cat. N. M0613, Dako) was diluted 1:250. Antigen retrieval and development was carried out using the fully automated (DAKO) instrument in accordance with manufacturers’ instructions.

Chor-IN-1 cells were detached from plates using 1% trypsin, washed twice with PBS and then fixed using 10% PBS-buffered formalin. After fixation, cells were included in 300 µl of pre-heated (60 °C) 2% agar solution (cat: 50005, Lonza). After agar solidification, cells were included in paraffin according to standard procedures. IHC was done with 3-µm cell block sections. Brachyury and EMA IHC were performed as described above.

IHC images have been scanned with Aperio Scan Scope XT (Aperio Technology). Length indicators are reported below. Magnification is 40× for all images. Digital zoom and scale bar are as follows: H&E (7.6×, 300); brachyury (33.2×, 60); EMA (24.8×, 80); physaliferous morphology (37.2×, 60); S-100 (21.2×, 100); vimentin (19.2×, 200); brachyury in cell line (14.8×, 200); EMA in cell line (11.2×, 200).

### Karyotype

Karyotype was obtained according to standard procedures: chromosome preparations were obtained from semi-confluent petri at the seventh passage by ipotonic treatment (KCl, HEPES, EDTA) followed by Cornoys fixative (Methanol:Acetic Acid = 3:1). Slides were G banded with Wright staining and analyzed at 100 X magnification with a Leica DM6000D microscope.

### Flow cytometry analysis of chordoma-specific surface antigens

U-CH2 and Chor-IN-1 cells were collected by trypsinization and recovered in their culture medium at 37 °C and 5% CO_2_ for 30 minutes, then washed with PBS containing 1% FCS (staining buffer) and counted. 0.5 × 10^6^ cells of each cell line were resuspended in 100 µl staining buffer and stained with 10 µl PE-conjugated mouse anti-human CD24 or its correspondent PE-conjugated isotype control (BD Biosciences, San Jose, CA, USA) for 20 minutes at room temperature. Samples were washed with staining buffer, fixed with 1% formaldehyde for 10 minutes at 37 °C and permeabilized with 90% methanol for 30 minutes on ice. After washing with staining buffer, 10 µl of FITC-conjugated mouse anti-cytokeratin (CAM5.2) or its correspondent FITC-conjugated isotype control (BD Biosciences, San Jose, CA, USA) were added and incubated for 20 minutes at room temperature. Samples were washed with staining buffer, then acquired and analyzed with a FACSCalibur cytometer and CellQuest software (BD Biosciences, San Jose, CA, USA). Analysis was performed on 10,000 events, gating out debris and doublets.

### DNA extraction

Genomic DNA was isolated from all chordoma cell lines and from frozen primary parental tumor tissue using NucleoSpin Tissue kit (Macherey-Nagel, Düren, Germany), according to manufacturer’s instructions.

DNA from patient-derived FFPE clinical samples for the Chor-IN-1 parental tumor and adjacent normal tissues was extracted from 7 µm slices using the RecoverAll Total Nucleic Acid Isolation kit (Thermo Fisher Scientific, Waltham, MA, USA), after tumor area selection and deparaffinization with xylene and absolute ethanol, according to manufacturer’s instructions. DNA yield and purity were determined by absorption measurement at 260 nm and by evaluation of the 260/280 ratio value using a Nanodrop 1000 Spectrophotometer (Thermo Fisher Scientific, Waltham, MA, USA).

### RNA extraction

RNA was extracted from the cell lines using the RNeasy Mini Kit (Qiagen, Hilden, Germany), as per manufacturer’s instructions. RNA yield and purity were evaluated by measurement of UV absorption at 260 nm and analysis of the 260/280 ratio using a Nanodrop 1000 Spectrophotometer (Thermo Scientific, Waltham, MA, USA).

### Short Tandem Repeat (STR) analysis

The STR identity profiles of all the chordoma cell line samples and of the Chor-IN-1 original tumor tissue were verified using the PowerPlex 16 HS System kit (Promega, Madison, WI, USA) which allows the simultaneous amplification of 15 STR loci, together with the amelogenin gender marker.

Briefly, 0.5 ng of purified genomic DNA was amplified with the recommended PCR protocol and capillary electrophoresis was performed using the 3100 Genetic Analyzer (Thermo Fisher Scientific, Waltham, MA, USA) according to the PowerPlex 16 HS System kit manufacturer’s guide.

Resulting data were processed using the GeneMarker HID v 2.4.0 software (Soft Genetics, State College, PA, USA) and comparative analysis was performed with the CLIFF (Cell Line Identity Finding by Fingerprinting) software^33^.

### End-point PCR and Sanger sequencing

The DNA polymorphism (c.G530A, p.G177D) in the “T” gene (Brachyury) was characterized by PCR amplification using the following primer pairs: FW 5′-TCACCAACAAGCTCAACGGA-3′; REV 5′-AAGCAGTCACCGCTATGAAC-3′ (1 cycle: 95 °C for 1 min; 30 cycles: 95 °C for 30 sec, 53 °C for 30 sec, 72 °C for 1 min; 1 cycle: 68 °C for 5 min) in a total reaction volume of 50 µl, containing 1× PCR buffer, 0.2 mM each dNTP mix, 0.8 µM of each primer, 1.25 units of FideliTaq DNA Polymerase (Affymetrix, Santa Clara, CA, USA) and 100 ng of genomic DNA as template.

Primers for PCR amplification and sequencing were designed using the freely available Primer3 software (http://primer3.ut.ee/) and synthesized using an Applied Biosystems 3900 Synthesizer (Thermo Fisher Scientific, Waltham, MA, USA).

For direct sequencing, the obtained PCR product was electrophoresed in agarose gel, then purified using NucleoSpin Gel and PCR clean-up (Macherey-Nagel, Düren,Germany), according to the manufacturer’s protocol, and subjected to Sanger sequencing with a ABI 3100 Genetic Analyzer instrument (Thermo Fisher Scientific, Waltham, MA, USA) with the same primers used for PCR amplification.

### Next Generation Sequencing (NGS) characterization using TruSight One (Illumina)

Chordoma clinical samples and cell lines were profiled using TruSight One (TSO) sequencing panel kit (Illumina, San Diego, CA, USA), following manufacturer’s instructions. The protocol allows the detection of single nucleotide variants and short InDels over the entire coding sequence of 4,813 genes (http://www.illumina.com/products/trusight-one-sequencing-panel.html). Briefly, 50 ng of input DNA were used for each sample for library preparation. Pooled libraries were quantified with the Qubit 2.0 Fluorometer System using the Qubit dsDNA HS assay kit (Thermo Fisher Scientific, Waltham, MA, USA). The libraries were sequenced in paired-end on the MiSeq platform (Illumina, San Diego, CA, USA) using the v3 reagent kit (2 × 150 bp). A minimum mean coverage depth of 100× was achieved for all the profiled samples.

The FASTQ files obtained from the instrument were analyzed following GATK gold standard procedures. Briefly, the sequences were mapped against the human reference genome (hg19) using BWA (v. 0.7.7) and the gene variants were identified using the Unified Genotyper (GATK v1.6) variant caller. Synonymous variants, variants with minor allele frequency >2% (reported in dbSNP) and low coverage variants (DP < 20, ADV < 7) were excluded from further analysis. Somatic variants characterizing the Chor-IN-1 cell line were also excluded by identifying variants in the normal tissue counterpart and subtracting them from the intersection of Chor-IN-1 cell line variants with the original tumor variants. A pairwise similarity matrix was computed comparing the variants identified in each chordoma cell line.

### Copy number analysis by NGS

In each genomic interval, coverage values obtained from TSO analysis were used to calculate the log2 ratio of the local versus sample mean coverage, imposing a cutoff of log2 > 0.2 for the regions of gain and of log2 < −0.2 for the regions of loss. Using TSO coverage data from all the analyzed chordoma cell lines, a consensus profile was created. For each genomic interval, we counted how many cell lines show a gain or loss in their profile and then plotted the percentage of cell lines showing that chromosomal aberration on the y-axis. The profile of the 50 chordoma clinical samples was obtained from the Progenetix database22; www.progenetix.org.

### Copy number analysis by aCGH

The Array Comparative Genomic Hybridization (aCGH) characterization was performed using the oligonucleotide-based microarray CytoChip Cancer 8 × 60 K v2.0 for Chor-IN-1, U-CH1 and U-CH2 samples and CytoChip ISCA 8 × 60 K v2.0 for MUG-Chor1 and JHC7 samples (BlueGenome Ltd, Cambridge, UK). Both arrays consist of 60,000 60-mer oligonucleotides spaced at about 50 kb density over the full genome. Labeling and hybridization were performed following the manufacturer’s protocol. 500 ng of the DNA extracted from each sample was co-hybridized with an equal amount of reference DNA. Reference sample was chosen according to the gender of the analyzed sample (Human Genomic DNA male G1471; Human Genomic DNA female G1521–Promega, WI, USA). The analysis was performed using InnoScan 710 Microarray scanner (Innopsys, Carbonne, France) and Mapix software (Innopsys, Carbonne, France). Genomic profiles were obtained using Blufuse Multi v4.3 software (BlueGnome Ltd, Cambridge, UK).

### TREx NGS-targeted kinase sequencing

A custom panel interrogating 487 human kinases was selected from the available pre-designed assays for TruSeq Targeted RNA expression kit (TREx Illumina, San Diego, CA, USA) using the Design Studio software (Illumina, San Diego, CA, USA). Custom TREx is based on amplicon technology generating PCR products with an average insert size between 70 and 80 bp. Library preparation was performed starting from 200 ng RNA input. To assess RNA quality, the RIN parameter (RNA Integrity Number) was evaluated using 2100 Bioanalyzer instrument, RNA 6000 nano kit (Agilent Technologies, Santa Clara, CA, USA). All the analyzed samples had a RIN value >8. The libraries were sequenced in single-end on MiSeq platform (Illumina, San Diego, CA, USA) with sequencing reagent kit v3 (1 × 50 bp). Fastq files were aligned to the human reference genome (hg19) using STAR (v. 2.5.1b)^34^. Raw count quantification was performed using Bedtools Coverage tool (v. 2.22.0)^35^, starting from the bam files. Normalization was performed using DESeq. 2 (v. 1.12.4)^36^ with default parameters and data were log2 transformed. Due to the similar length of the produced fragments, a length normalization step was not performed. R was used to calculate the distance matrix (distance method = maximum) between the kinases in the chordoma cell lines and “complete linkage” clustering was applied (hclust function) to generate 3 groups: high, low and medium expressed kinases. These clusters were then used to generate a plot of kinase expression pattern in chordoma cell lines by means of Kohonen maps, used for visualization.

### Real Time quantitative PCR

Real Time quantitative PCR (RT-qPCR) was carried out using SYBR green technology; specific primers were designed for the genes of interest using the freely available Primer3 software (http://primer3.ut.ee/) and synthesized using an Applied Biosystems 3900 Synthesizer.

RT-qPCR data were obtained using TaqMan Reverse Transcription Reagents (Thermo Fisher Scientific, Waltham, MA, USA) and random hexamer priming to reverse transcribe RNA to complementary DNA (cDNA), according to manufacturer’s instructions. Real Time quantitative PCR (qPCR) was carried out on a ABI Prism 7900HT Applied Biosystems Sequence Detector (Thermo Fisher Scientific, Waltham, MA, USA), with reagents and materials from Applied Biosystems/Thermo Fisher Scientific (Power SYBR® Green PCR Master Mix), according to manufacturer’s instructions, in a volume of 12.5 μl per reaction, each containing approximately 10–12 ng cDNA (diluted in TE buffer 1×), 300 nM primers for the different targets and for 3 endogenous reference controls (glucuronidase beta (GUSB), peptidylprolylisomerase A (PPIA), and 18S ribosomal RNA), as detailed in Suppl. Table 2. Each sample was assayed in duplicate qPCR reactions. Relative quantification of expression levels was calculated following the manufacturer-suggested ΔΔCt method using the average of the 3 above reference controls^37^.

### Data Availability

The NGS data reported in the submitted manuscript have been deposited in the BioProject database with the following ID: PRJNA382303, and will be released upon paper acceptance.

## Electronic supplementary material

Supplementary Information
