# Supplementary material for: Establishment and genomic characterization of the new chordoma cell line Chor-IN-1
Source: Sci Rep. 2017 Aug 23;7:9226. doi: 10.1038/s41598-017-10044-3 (PMC5569021; doi:10.1038/s41598-017-10044-3)
Supplement: Supplementary file 1 — Supplementary Information [file 41598_2017_10044_MOESM1_ESM.pdf]

Supplementary Information

Establishment and genomic characterization of the new chordoma cell line Chor-IN-1

Roberta Bosotti[1§]*, Paola Magnaghi[1§], Sebastiano Di Bella[1], Liviana Cozzi[1], Carlo Cusi[1], Fabio Bozzi[2], Nicola Beltrami[3], Giovanni Carapezza[1], Dario Ballinari[1], Nadia Amboldi[1], Rosita Lupi[1], Alessio Somaschini[1], Laura Raddrizzani[1], Barbara Salom[1], Arturo Galvani[1], Silvia Stacchiotti[2], Elena Tamborini[2] & Antonella Isacchi[1]

[1] Oncology, Nerviano Medical Sciences, Nerviano (MI), Italy

[2] Fondazione IRCCS Istituto Nazionale dei Tumori, Milan, Italy

[3] L.C. Laboratori Campisi, Avola (SR), Italy

[§] These authors equally contributed to the work

*corresponding author:

Roberta Bosotti

Oncology

Nerviano Medical Sciences

Viale Pasteur 10

20014 Nerviano (MI)

Email: roberta.bosotti@nervianoms.com

Phone: +39-0331581286

Supplementary Figures:

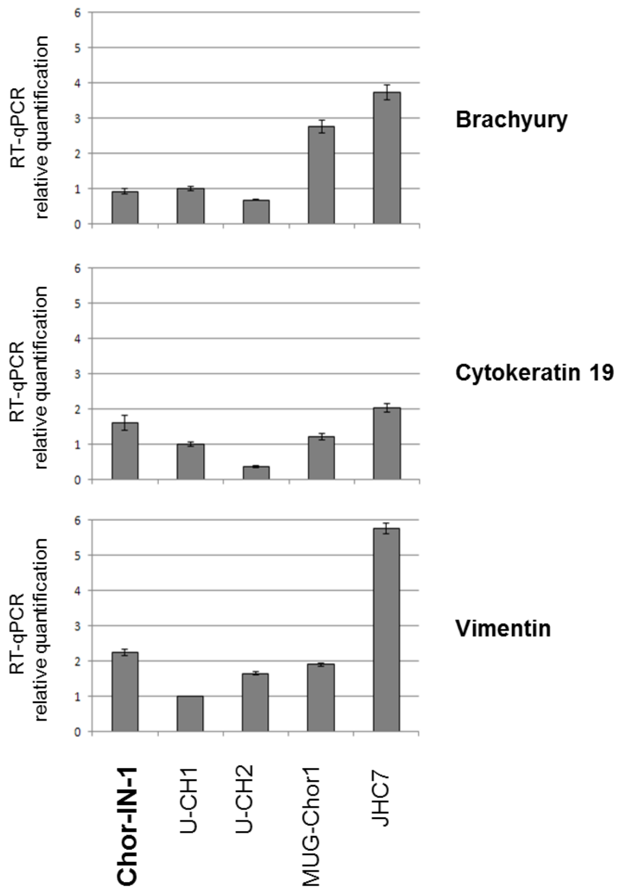

Supplementary Figure 1: **RT-qPCR analysis of brachyury, cytokeratin 19 and vimentin gene expression in Chor-IN-1 and in a panel of chordoma cell lines**.

Histogram bars represent RT-qPCR relative quantification results, carried out as described in M&M and normalized using U-CH1 as a reference sample.

	AMEL		CSF1PO		D13S317		D16S539		D18S51		D21S11		D3S1358		D5S818		D7S820		D8S1179		FGA		TH01		TPOX		vWA		PentaD		PentaE	
**Chor-IN-1 cell line (established in house)**	X	Y	10	12	11	12	13	13	15	17	31	31.2	17	18	9	12	8	9	12	15	21	22	9.3	9.3	7	8	18	18	13	13	12	21
**Chor-IN-1 original tumor**	X	Y	10	12	11	12	13	13	15	17	31	31.2	17	18	9	12	8	9	12	15	21	22	9.3	9.3	7	8	18	18	13	13	12	21
**U-CH1 cell line (Chordoma Foundation)**	X	Y	10	11	11	13	12	13	15	15	28	29	15	15	11	12	9	12	10	15	20	21	7	7	8	11	17	17	11	11	7	10
**reference profile (DSMZ_DB)**	X	Y	10	11	11	13	12	13							11	12	9	12					7	7	8	11	17	17				
**U-CH2 cell line (Chordoma Foundation)**	X	X	11	12	11	11	12	12	12	18	29	30	17	17	10	11	8	12	13	13	21	22.2	6	6	8	8	17	17	12	13	12	15
**reference profile (COSMIC_DB)**	X	X	11	12	11	11	12	12							10	11	8	12					6	6	8	8	17	17				
**MUG-Chor1 cell line (ATCC)**	X	X	11	11	11	11	11	14	17	23	29	33.2	14	17	11	12	8	11	11	12	21	26	9.3	9.3	8	8	15	15	13	13	5	12
**reference profile (DSMZ_DB)**	X	X	11	11	11	11	11	14							11	12	8	11					9.3	9.3	8	8	15	15				
**JHC7 cell line (Chordoma Foundation)**	X	X	11	11	11	11	11	11	12	12	27	31.2	17	17	13	13	7	10	13	14	21	23	6	8	10	11	17	17	6	11	15	15
**reference profile (ATCC_DB)**	X	X	11	11	11	11	11	11							13	13	7	10					6	8	10	11	17	17				

Supplementary Figure 2: Characterization of chordoma cell lines by STR profiling

Chordoma cell line STR profiles are compared against the corresponding public reference STR profile (dark grey). The profile obtained for the newly established Chor-IN-1 cell line and Chor-IN-1 original tumor is highlighted in light grey.

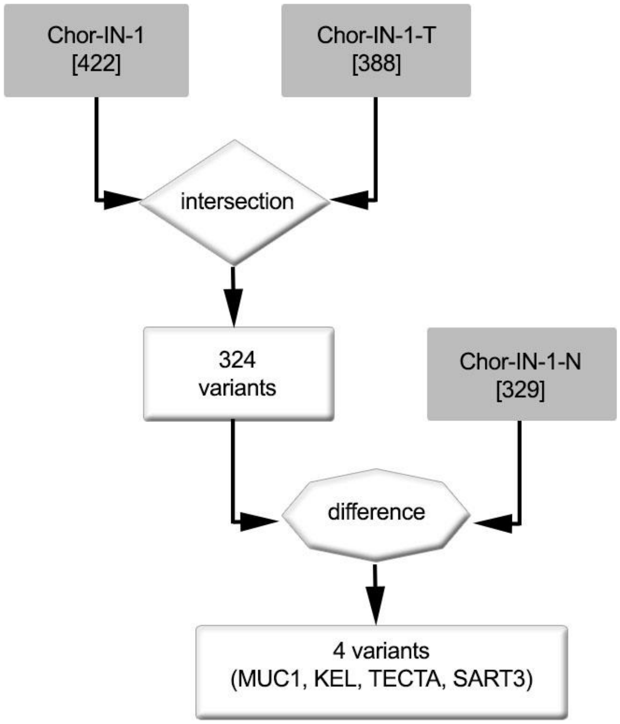

Chr	Start	End	Ref	Alt	Gene	Func.refGene	ExonicFunc.refGene	AAChange
chr1	155162090	155162090	G	T	MUC1	exonic	nonsynonymous SNV	p.P24T
chr7	142638362	142638362	A	-	KEL	exonic	frameshift deletion	p.S726fs
chr11	121028593	121028593	G	A	TECTA	exonic	nonsynonymous SNV	p.R1450H
chr12	108926001	108926001	C	T	SART3	exonic	nonsynonymous SNV	p.E556K

Supplementary Figure 3: **Flowchart of the identification of somatic variants in Chor-IN-1**

Flowchart summarizing the procedure followed for the identification of somatic gene variants in Chor-IN-1. The intersection of variants identified in the Chor-IN-1 cell line and in the corresponding tumor sample yielded 324 common variants (~80%). Germline variants were then subtracted, resulting in the identification of 4 gene variants, as detailed in the lower panel. Chor-IN-1-T and Chor-IN-1-N indicate tumor and normal tissue samples, respectively.

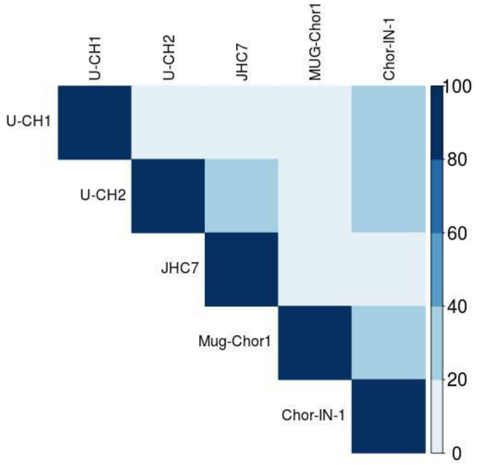

Supplementary Figure 4: **Comparison of the number of variants identified in chordoma cell lines**

Intersection of the gene variants identified in different chordoma cell lines. Light to dark blue shades in squares represent the percent number of variants in common between each cell line and all the others.

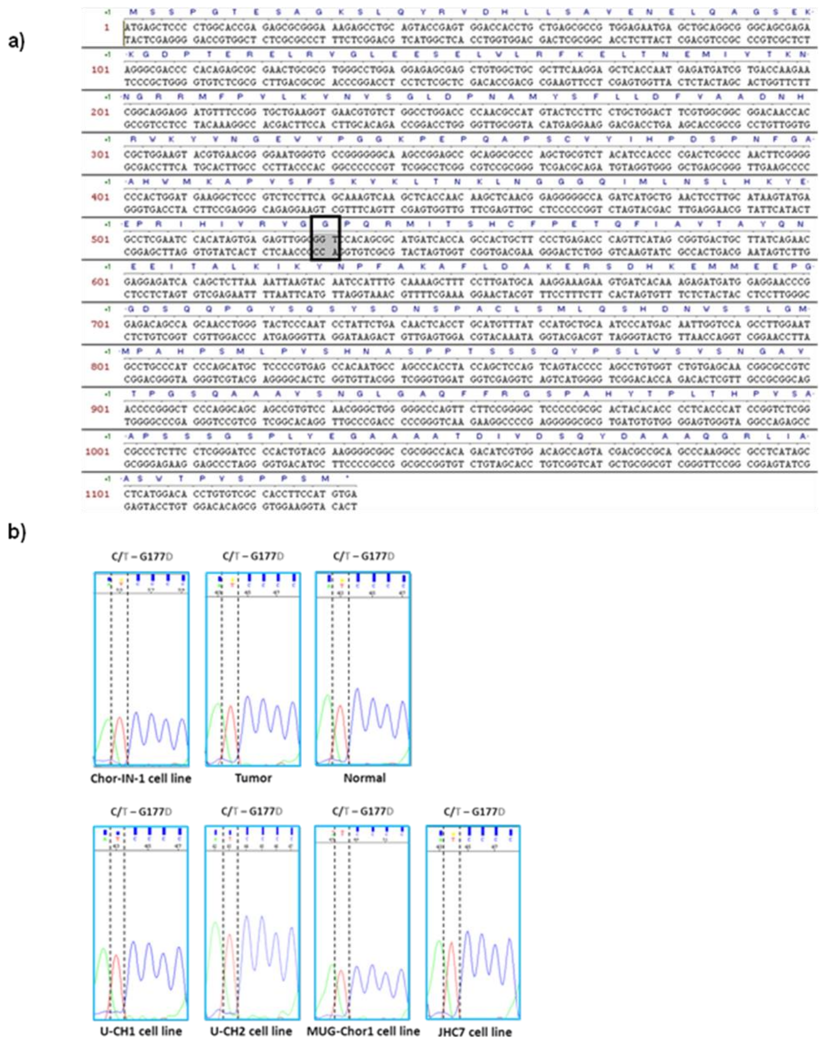

Supplementary Figure 5: **Brachyury p.G177D polymorphism**

a) The DNA sequence and the corresponding protein translation of brachyury (T) is reported. The position of c.530G>A (p.G177D) polymorphism is boxed. **b**) Sanger sequencing electropherograms show the c.530 nucleotide position in the following samples: Chor-IN-1 cell line, original tumor, adjacent normal sample (upper panel); U-CH1 cell line, U-CH2 cell line, MUG-Chor1-1 cell line and JHC7 cell line (lower panel).

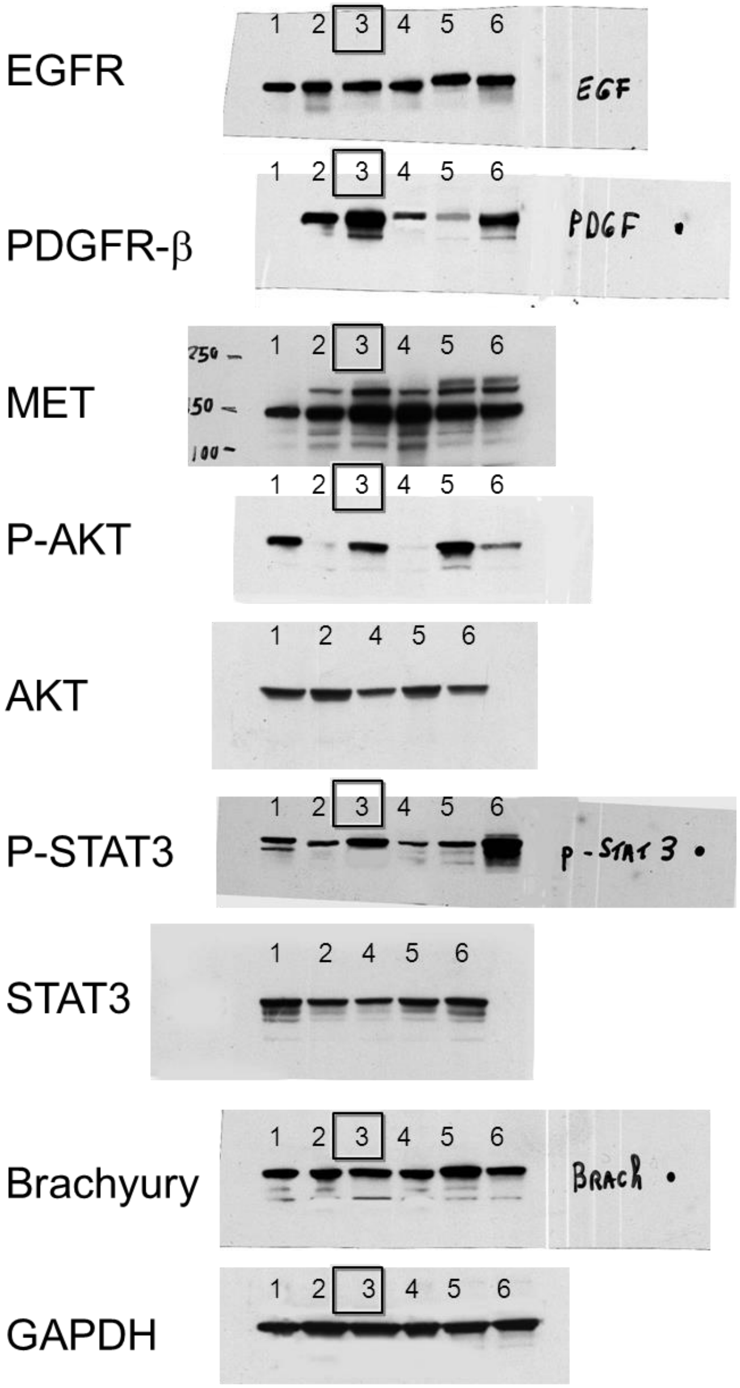

Supplementary Figure 6: **Full-length blots reporting the molecular characterization of Chor-IN-1 cell line**

The original blots included also an additional lane (number 3) reporting the U-CH2 cell line obtained from a different provider (ATCC). This lane was removed from Fig.2 because this cell line did not undergo genomics characterization.

Supplementary Tables

Supplementary Table 1: **Kinase gene expression in chordoma cell lines. Values are reported as log2(normalized counts).**

Kinase	Chor-IN-1	JHC7	Mug-Chor-1	U-CH1	U-CH2
ABL1	12.82	11.26	12.84	12.67	11.89
ABL2	12.18	12.03	11.34	12.69	12.31
ACVR1	10.23	11.52	11.42	11.76	12
ADCK2	11.21	10.66	11.58	11.22	11.68
ADCK3	10.28	10.18	9.67	11.3	9.45
ADCK4	10.12	10.49	11.77	10.45	9.38
ADRBK1	11.85	12.49	12.01	11.11	11.85
AKT1	13.64	12.4	12.78	13.34	13.39
AKT2	12.43	12.98	13.01	12.36	13.77
AKT3	12.72	13.43	13.16	10.11	12.94
ALPK2	12.78	12.33	12.25	12.37	12.61
ARAF	13.55	13.27	12.91	13.44	13.31
ATM	10.87	11.53	11.31	10.45	10.63
ATR	10.49	9.97	11.3	11.07	10.91
AURKA	12.8	12.89	12.13	13.19	12.15
AURKB	11.41	10.2	9.56	11.12	9.57
AXL	15.59	9.5	13.88	13.5	15.93
BCKDK	12.84	13.04	12.78	12.78	12.88
BMP2K	11.22	12.56	11.02	11.03	11.69
BMPR2	12.37	12.85	12.46	12.67	12.48
BRD4	13.06	13.59	13.32	12.81	13.34
BUB1B	11.58	10.96	11.94	11.67	11.4
CAMK1	11.5	11.24	11.24	10.55	9.86
CAMK2D	12.28	13.19	12.5	12.46	13.14
CAMKK2	11.52	10.52	11.02	10.78	10.94
CDC42BPA	12.65	12.43	12.19	12.66	12.44

CDC42BPB	13.56	12.61	11.99	13	13.1
CDK11A	9.59	11.67	11.59	10.63	10.27
CDK11B	11.47	12.77	11.94	11.94	11.45
CDK12	11.83	11.93	12.13	12.33	11.45
CDK13	11.46	10.56	11.41	11.43	11.5
CDK14	11.59	9.19	10.73	9.26	10.51
CDK16	12.82	14.01	13.24	13.23	13.17
CDK17	11.84	10.65	11.01	11.57	11.48
CDK18	11.66	13.02	12.73	11.06	11.61
CDK19	11.42	11.18	9.81	11.11	9.97
CDK2	11.99	10.54	11.36	11.78	11.09
CDK4	13.21	12.42	13.61	13.64	13.77
CDK5	12.36	11.56	12.13	11.55	12.76
CDK6	13.22	11.57	11.78	13.09	13.15
CDK7	14.24	12.88	13.83	13.93	13.65
CDK9	11.97	11.23	12.36	12.71	10.83
CHEK2	9.96	10.2	10.24	10.23	10.7
CHUK	11.6	10.37	10.41	11.3	11.25
CLK1	10.06	11.77	11.85	11.06	10.4
CLK2	10.5	10.93	11	11.31	10.3
CLK3	10.07	9.43	10.34	9.82	10.26
CSK	11.68	9.93	11.65	10.28	12.07
CSNK1A1	13.09	13.08	13.14	13.11	12.89
CSNK1D	12.31	12.63	12.26	12.62	12.59
CSNK1E	12.72	12.73	11.79	13.01	12.89
CSNK1G2	10.89	10.83	11.22	10.62	11.2
CSNK1G3	11.42	11.86	10.82	11.48	11.19
CSNK2A1	12.57	12.94	12.42	12.71	11.9
CSNK2A2	11.17	10.83	11.19	10.41	11.84
DAPK3	10.24	11.38	10.68	11.49	11.14
DCLK2	10.05	10.24	11.39	10.95	10.95
DDR1	13.58	12.48	12.62	13.37	13.05
DDR2	12.51	12.7	13.53	11.5	13.12
DYRK1A	11.81	11.45	11.98	11.9	11.62
DYRK1B	10.46	8.11	10.88	9.97	11.43
DYRK4	12.14	12.21	10.93	11.49	12.61
EGFR	11.37	11.59	11.29	12.66	11.36
EIF2AK1	14.15	13.95	14.06	14.62	14.45
EIF2AK2	12.27	12.98	12.48	12.54	12.13
EIF2AK4	12.36	12.03	12.49	12.64	13.05
EPHA4	9.09	11.33	9.94	11.55	8.53

EPHB4	11.58	10.8	10.97	11.72	11.57
FASTK	12.09	11.98	12.11	12.07	12.59
FER	11.7	9.83	10.38	10.75	10.19
FGFR1	13.44	8.2	13.32	11.25	12.49
FLT1	8.7	12.92	13	11.56	9.69
GAK	11	10.37	11.62	11.22	10.96
GRK6	11.62	12.68	11.9	8.92	11.78
GSK3A	12.21	12.58	12.94	12.16	13.24
GSK3B	10.86	10.78	10.95	11.32	11.33
HIPK1	11.51	11.97	12.29	12.7	11.86
HIPK2	10.06	11.54	9.85	10.33	11.64
HIPK3	12.89	13.62	12.75	11.06	12.4
HUNK	10.85	12.36	13.28	13.47	10.31
IGF1R	11.11	10.3	10.62	7.59	11.28
IKBKAP	11.86	11.03	11.6	11.74	10.38
IRAK1	12.68	12.22	12.55	12.59	13.24
IRAK4	10.83	9.85	11.39	11.23	11.06
ITK	10.33	10.64	10.42	11.28	9.2
JAK1	10.65	13.07	12.02	11.72	11.16
JAK2	10.24	9.91	9.68	11.28	9.47
LATS2	11.88	11.77	12.39	11.91	13.12
LIMK1	10.75	10.37	10.92	11.43	12.91
LIMK2	10.82	9.41	10	9.35	12.05
LYN	10.54	10.3	11.1	10.94	10.52
MAP2K1	12.78	11.87	12	12.8	12.53
MAP2K2	13.45	13.76	13.29	13.21	13.87
MAP2K3	11.52	12.41	11.64	12.35	11.12
MAP2K5	10.85	9.96	10.47	10.5	11.22
MAP3K10	9.75	10.63	10.42	10.68	11.41
MAP3K2	12.89	12.42	12.1	12.48	12.68
MAP3K3	12.95	11.96	11.6	11.01	12.06
MAP3K4	11.17	13.08	9.97	9.69	11.75
MAP3K6	9.9	10.87	10.61	10.17	10.14
MAP3K7	11.94	12.28	10.9	11.83	11.03
MAP4K2	9.91	11.22	10.96	10.3	10.04
MAP4K4	11.5	11.95	10.9	12.05	12.3
MAP4K5	11.69	12.06	11.17	11.76	12.45
MAPK1	12.08	13.13	11.53	11.72	12.42
MAPK11	9.87	9.18	10.32	10.63	10.72
MAPK12	10.5	11.45	11.2	10.92	11.6
MAPK14	12.45	12.63	12.24	12.67	11.6

MAPK3	12.33	11.47	12.21	11.74	12.83
MAPK6	12.31	12.04	12.12	12.84	12.88
MAPK7	10.77	10.91	11.09	11.96	10.31
MAPK9	11.96	12.43	11.35	11.9	11.69
MAPKAPK2	13.11	13.44	12.5	12.1	13.29
MARK3	12.49	11.91	11.58	12.77	12.71
MAST3	9.64	11.16	10.84	10.17	10.9
MELK	10.86	10.45	10.53	10.91	10.88
MET	15.1	12.91	13.09	14.07	14.41
MINK1	12.7	10.81	10.62	12.3	10.93
MKNK1	10.63	11.34	10.35	10.79	9.28
MKNK2	13.2	14.4	12.6	12.1	13.45
MLKL	12.21	10.74	10.45	11.53	10.98
MST4	11.3	11.29	10.85	10.07	10.83
MTOR	10.84	12.32	11.04	11.15	10.83
NEK2	11.95	12.21	11.45	11.59	11.38
NEK6	10.49	10.41	11.01	11.07	11.31
NEK7	15.36	13.57	12.89	13.75	16.71
NEK9	12.17	8.34	11.61	10.84	11.85
NLK	10.88	12.22	10.17	11.04	11.48
NPR2	11.1	9.16	10.21	10.46	10.33
NRBP1	12.41	12.49	12.4	12.31	12.24
NRBP2	10.75	11.08	11.17	10.1	10.36
NUAK1	11.57	9.2	11.19	10.04	9.95
OXSR1	13.55	11.72	13.09	12.59	11.66
PAK2	12.08	12.16	12.42	12.5	12.74
PAK4	11.81	11.45	11.86	11.78	12.54
PBK	13.43	11.45	12.78	12.22	10.98
PDK1	8.95	10.7	9.45	11.07	8.79
PDK3	8.94	11.72	10.72	12.23	8.76
PDPK1	11.97	10.92	11.6	10.98	10.95
PEAK1	11.16	9.33	11.55	10.81	11.59
PHKG2	11.01	11.31	11.28	11.66	10.54
PIK3R4	9.99	9.92	10.94	10.19	10.61
PIM1	9.21	10.79	10.12	10.66	9.58
PIM2	11.05	11.72	11.04	12.37	10.91
PIM3	10.58	11.38	11.97	11.73	12.77
PINK1	11.54	14.12	11.3	12.21	12.99
PKN1	12.44	13.23	12.24	11.45	12.95
PKN2	11.15	10.9	11.95	10.87	10.5
PLK1	13.67	12.43	12.43	12.81	11.73

PLK2	15.36	11.35	14.55	16.04	11.69
PRKAA1	11.41	11.28	11.79	11.37	11.45
PRKACA	13.26	12.48	12.69	12.61	13.04
PRKACB	10.6	10.52	10.29	10.78	10.45
PRKAG1	13.61	12.79	13.31	12.83	13.45
PRKCD	11.28	9.78	12.27	10.41	10.23
PRKCI	10.65	10.84	10.48	10.82	10.8
PRKCZ	11.05	13	12.47	8.96	11.7
PRKD3	11.56	11.29	11.53	11.71	11.37
PTK2	12.85	12.66	12.31	11.78	12.03
PTK2B	11.91	9.33	11.34	9.52	10.31
PXK	10.24	9.58	11.31	10.38	10.77
RAF1	12.51	13.15	13.08	12.4	11.65
RIOK1	11.17	11.73	10.2	11.35	10.18
RIOK2	11.36	11.84	11.54	11.56	11.87
RIOK3	12.55	12.1	12.66	12.72	11.63
RIPK2	11.58	12.33	10.76	12.25	11.8
ROCK1	12.01	11.98	12.02	11.25	12.05
ROCK2	12.12	12.03	12.08	11.36	11.8
RPS6KA1	9.77	11.83	11.15	10.63	11.01
RPS6KA3	11.38	11.63	12.19	12.02	12.52
RPS6KB1	10.57	10.95	10.98	10.94	10.49
RPS6KB2	10.99	11.52	11.14	10.57	11.28
RPS6KC1	9.5	10.58	9.9	11.51	9.9
RYK	11.42	11.68	12.35	11.84	12.62
SCYL1	12.43	12.88	12.94	12.15	12.91
SCYL2	11.85	10.92	11.35	12.19	11.69
SGK1	13.86	12.85	11.38	13.59	14.41
SGK223	12.48	11.4	12.03	9.64	11.71
SIK3	12.23	12.14	12.55	10.37	11.86
SLK	12.55	10.74	11.61	11.51	11.47
SMG1	10.97	10.93	10.83	10.72	10.14
SRC	10.18	11.22	10.58	11.44	10.64
SRPK1	12.25	12.56	11.27	12.24	10.63
SRPK2	10.5	11.17	10.9	10.21	10.77
STK10	13.06	12.3	11.76	11.67	11.36
STK16	10.62	10.53	11.26	10.68	10.05
STK17A	13.15	11.88	10.92	13.08	12.84
STK17B	11.26	12.69	12.01	12.31	11.22
STK19	11.54	12.21	11.05	12.08	10.92
STK24	14.75	12.98	13.26	12.64	12.01

STK25	12.59	13.39	13.73	14.43	12.76
STK36	9.88	10.72	11.29	11.05	9.18
STK38	13.2	12.68	12.41	13.43	12.25
STK38L	10.62	10.11	9.44	8.78	12.6
STK39	11.28	11.63	11.12	12.34	12.09
STK4	10.8	11.75	11.02	11.52	10.32
STK40	10.91	12.87	11.56	11.38	11.93
STRADA	10.57	10.42	10.74	10.81	10.74
STRADB	11.95	12.84	11.31	12.42	11.35
TAOK1	12.2	12.13	11.81	12.54	12.23
TAOK2	10.61	9.67	10.63	10.3	11.16
TAOK3	11.77	9.9	11.17	10.12	11.38
TBK1	12.23	11.44	12.55	12.43	11.95
TESK1	11.22	10.92	10.98	11.32	10.85
TGFBR1	14.18	11.49	12.93	14.79	14.91
TGFBR2	11.82	12.34	11.36	10.71	11.03
TLK1	10.47	11.14	10.97	11.6	11.16
TRIB3	10.63	13.44	11.44	7.35	12.92
TRIM28	14.28	14.86	15.25	14.18	13.53
TRIO	11.65	11.78	10.19	12.25	11.83
TRPM7	11	10.17	11.53	10.91	11.08
TTBK2	10.44	10	10.45	10.4	10.7
TWF1	13.11	12.19	12.73	13.21	13.3
TYK2	11.35	12.14	11.76	11.74	11.96
TYRO3	10.7	9.34	9.92	10.39	10.37
UHMK1	14.01	13.83	13.21	14.16	13.6
ULK1	9.82	9.61	10.28	10.15	10.54
ULK3	12.27	11.16	12.92	11.61	12.69
VRK1	11.94	10.58	11.25	11.12	11.1
VRK2	11.9	12.34	11.57	11.45	11.87
WEE1	12.22	12.89	12.73	11.31	11.26
WNK1	14.26	11.66	11.07	11.18	13.42
YES1	12.81	13.88	12.74	12.75	12.45
ZAK	10.47	12	11.71	12.61	13.36
ACVR1B	8.56	8.18	8.4	8.8	9.07
ACVR1C	4.69	9.37	4.87	8.81	6.88
ACVR2B	8.9	9.58	8.9	8.35	7.79
ACVRL1	6.1	4.38	2.65	6.13	5.57
ADCK1	7.81	6.61	6.95	7.12	7.96
ADCK5	9.61	9.13	9.21	9.3	8.9
ADRBK2	5.69	7.73	7.16	8.2	10.24

ALPK1	7.68	7.57	8.54	8.55	9.08
BCR	11.01	10.68	10.47	10.36	10.96
BMPR1A	11.16	10.59	10.31	9.92	10.77
BRAF	9.78	10.51	10.44	9.42	10.71
BRSK1	6.13	9.84	8.43	9.23	8.68
CAMK1D	6.04	10.55	11.2	8.55	10.34
CAMK2B	5.1	6.97	8.87	8.88	5.47
CAMK2G	9.44	9.06	8.43	9.53	10.54
CAMKK1	8.8	7.27	7.9	8.99	7.75
CASK	10.42	10.86	9.33	10.22	10.35
CDC7	8.67	8.72	10.2	10.11	9.09
CDK10	9.59	8.67	9.87	11.09	9.69
CDK15	8.73	3.85	2.65	7.72	7.78
CDK20	9.89	9.07	9.91	11.02	9.34
CDK8	9.69	9.57	9.4	10.23	9.8
CDKL1	9.32	8.22	7.24	8.92	9.31
CDKL3	6.52	7.18	7.84	7.33	7.83
CDKL4	6.94	4.43	4.94	4.61	4.5
CHEK1	11	10.26	10.1	9.73	10.22
CIT	8.95	7.99	9.6	8.8	8.86
CLK4	9.64	10.59	10.48	9.81	9.59
CSNK1A1L	7.56	7.08	8.32	7.79	7.11
CSNK1G1	9.86	8.68	8.83	9.84	9.94
DAPK1	5.81	8.78	4.58	10.98	7.13
DAPK2	9.25	7.25	4.64	7.51	7.92
DMPK	9.4	8.43	9.97	9.17	9.52
DSTYK	10.03	10.33	9.51	9.98	9.7
EEF2K	10.12	9.83	10.15	9.38	9.48
EIF2AK3	9.98	11.01	10.13	10.15	10.24
EPHA2	8.97	9.95	7.8	9.52	9.87
ERBB2	9.07	8.38	8.7	8.46	8.74
ERN1	8.47	10.36	7.97	7.8	9.57
FGFR2	9.21	6.11	9.96	6.85	10.87
FRK	7.23	7.02	5.01	8.44	5.86
FYN	5.83	11.23	7.75	10.29	9.98
GRK5	9.67	10.64	8.63	9.56	9.27
GSG2	7.91	7.05	6.66	8.19	6.24
ICK	10	9.09	9.73	9.59	9.88
IKBKE	9.66	7.49	8.33	10.12	9.4
ILK	8.35	8.33	7.6	7.4	6.58
INSR	9.33	9.93	8.33	6.73	8.66

IRAK2	8.46	8.31	7.03	9.63	7.6
LATS1	10.12	10.49	9.47	10.1	10.05
LMTK2	9.11	9.65	9.5	9.57	10.11
LTK	6.38	9.34	6.16	3.55	6.06
MAK	6.07	8.23	6.53	9.1	4.36
MAP2K6	6.38	7.02	9.63	11.46	6.22
MAP2K7	8.82	8.82	8.6	8.47	8.53
MAP3K1	8.32	8.37	7.85	8.35	10.28
MAP3K11	7.65	7.94	8.55	8.13	7.74
MAP3K12	8.41	6.83	10.84	8.39	10.76
MAP3K13	6.65	8.55	7.89	9.63	8.56
MAP3K14	8.79	9.1	8.16	10.61	8.79
MAP3K5	11.48	9.12	4.11	7.88	7.48
MAP3K8	8.03	6.2	6.64	8.93	6.28
MAP3K9	8.01	7.7	6.74	7.92	7.26
MAP4K3	10.4	10.9	10.28	10.12	10.7
MAPK13	6.44	6.04	5.1	4.89	5.87
MAPK8	8.3	8.48	7.94	6.77	9.05
MARK1	8.67	9.16	9.55	10.54	9.07
MAST2	9.48	10.72	9.23	10.15	9.05
MAST4	9.24	8.52	10.63	8.08	9.59
MASTL	10.33	9.13	9.43	9.63	9.23
MOK	10.46	10.42	7.75	9.24	9.16
MYLK2	5.39	4.81	4.65	5.49	6.36
NEK1	9.68	9.61	9.46	9.71	9.56
NEK3	9.51	8.96	9.18	9.2	9.87
NEK4	10.27	9.34	10.41	9.88	9.02
NEK8	8.05	7.28	7.19	7.24	8.07
NUAK2	7.25	10.81	7.16	8.71	9.52
PAK1	10.85	9.9	9.77	10.34	10.84
PAN3	9.1	9.16	10.18	8.75	8.87
PASK	7.44	6.36	8.34	7.85	6.31
PDGFRB	7.89	9.11	8.06	5.83	10.45
PDK4	8.2	12.45	9.14	8.53	8.1
PKMYT1	9.52	7.63	9.02	9.11	8.39
PKN3	10.09	8.36	9.71	7.73	7.4
PLK3	9.26	10	8.21	8.88	10.55
PLK4	9.47	9.76	10.09	9.88	9.33
PRKCA	7.74	6.7	7.12	9.17	9.49
PRKCE	8.56	8.67	8.84	7.92	8.91
PRKD2	9.68	10.78	10.21	9.8	9.14

PRKDC	9.98	10	9.76	9.21	9.84
PRKX	8.77	10.85	8.86	12.16	4.46
PRPF4B	8.03	7.52	7.69	7.81	6.33
PSKH1	9.84	8.53	9.75	8.73	10.07
PTK7	8.24	8.73	8.6	5.72	8.27
RIPK1	10.26	10.01	10.31	9.88	8.88
RIPK4	10.38	9.87	11.22	8.58	9.62
RNASEL	9.1	8.32	9.25	9.48	9.12
ROR1	7.54	6.02	3.5	7.22	0.59
RPS6KA2	11.32	15.26	7.18	7.76	7.23
RPS6KA4	7.94	6.7	7.59	8.05	7.77
RPS6KA5	7.81	9.14	6.97	6.06	7.1
RPS6KL1	8.77	5.21	6.65	9.05	7.35
SCYL3	8.69	9.05	8.5	8.7	8.39
SIK1	7.57	7.75	6.39	8.75	7.52
SIK2	11.15	10.59	10.59	10.1	10.66
SNRK	9.41	8.88	9.5	8.83	8.32
STK11	9.71	10.07	10.27	9.77	9.99
STK3	10.35	11.08	9.83	10.4	10.2
STK32B	6.65	8.6	10.12	5.96	7.57
STK32C	8.67	7.23	7.83	7.87	7
STK33	10.33	9.63	10.11	0.7	8.59
STK35	9.22	9.59	9.77	9.61	8.32
STYK1	5.16	6.57	8.18	6.75	11.16
TAF1	9.52	9.89	9.7	9.72	9.6
TBCK	9.52	11	10.68	9.28	10.06
TEC	6.78	8.61	8.87	9.45	6.75
TEK	5.04	9.61	7.29	5.77	7.08
TESK2	8.06	10.58	8.65	4.67	7.25
TEX14	6.35	10.17	9.22	6.04	8.37
TNK2	7.59	6.06	8.61	7.25	7.31
TP53RK	9.67	8.42	10.08	10.37	9.68
TRIB1	9.46	10.03	10.13	9.76	11.07
TRIM24	10.43	10.2	9.59	8.92	10.77
TSSK1B	6.49	6.47	3.2	4.67	5.37
TSSK6	7.01	7.38	7.35	6.38	6.85
TTK	10.49	10.34	8.96	9.9	9.13
TTN	10.45	11.75	8.08	9.82	7.73
ULK2	10.83	10.59	10.17	9.89	9.35
ULK4	10.62	7.46	6.54	7.86	7.42
VRK3	10.15	10.48	10.05	9.32	10.23

WNK4	9.37	10.4	4.4	7.54	5.89
AAK1	2.75	3.5	2.44	3.47	1.97
AATK	1	5.05	5.92	3.07	3.92
ALK	0	1.78	0.96	0	1.98
ALPK3	4.65	3.8	4.85	3.48	6.58
AMHR2	1	5.51	4.26	5.13	1.01
ANKK1	0	4.45	4.94	3.94	2.68
AURKC	5.45	7.85	6.91	5.33	7.02
BLK	1.15	0.95	0	1.44	1.77
BMPR1B	0	1.57	3.36	4.22	4.7
BMX	0	1.94	0.61	0	1.53
BRSK2	3.26	6.68	6.24	5.15	3.07
BTK	1.44	4.45	2.77	0.93	1.53
C9orf96	2.44	2.66	3.47	3.24	2.54
CAMK1G	1	1.98	0.56	1.17	0
CAMK2A	3.61	5.15	5.87	4.16	6.81
CAMK4	2.85	0.6	6.76	7.42	7.18
CAMKV	5.33	5.53	3.03	0.93	1.58
CDC42BPG	1.96	2.53	5.07	1.06	3.83
CDKL2	5.43	5.55	7.43	0	5.33
CDKL5	2.63	6.42	5.7	2.03	1.57
CSF1R	2.62	2.29	1.53	2.93	6.26
DCLK1	1.32	5.13	7.43	5.49	4.96
DCLK3	0	0	0	0.7	0.56
EPHA1	1.21	2.14	1.3	0.54	6.22
EPHA3	1.89	1.8	0.61	5.21	6.32
EPHA5	0	3.24	5.69	3.85	0
EPHA6	0	3.29	0	2.23	0.56
EPHA7	0	4.81	5.39	1.06	0
EPHB1	1.86	7.56	6.6	4.14	6.4
EPHB2	1.21	5.84	2.19	8.04	7.01
EPHB3	0.93	2.42	7.06	3.9	5.39
EPHB6	2.46	1.94	7.15	7.15	7.98
ERBB3	7.58	6.74	6.23	5.8	4.54
ERBB4	0	4.37	2.15	5.02	1.57
ERN2	2.85	2.13	1.03	0.7	0
FES	7.09	2.79	1.36	0	6.32
FGFR3	1	7.51	10.3	5.46	6.02
FGFR4	2.38	9.34	4.5	4.69	7.67
FGR	1.64	5.67	3.67	2.72	2.15
FLT3	0	0.55	0.56	0.54	0.59

FLT4	0	0.99	6.87	2.46	0.56
GRK1	1.32	2	3.6	0.93	2.28
GRK4	5.01	2.42	3.96	5.46	4.44
GRK7	0	1.35	0	0	0
GUCY2C	0	0.99	0	0.54	0.59
GUCY2D	1	2.54	0	2.28	1.27
GUCY2F	0	0	0	0	0
HCK	1	5.15	0	2.09	8.25
HIPK4	5.6	4.52	4.07	2.71	5.34
IKBKB	4.97	3.62	4.19	5.2	3.57
INSRR	0	0.95	2.34	3.29	1.01
IRAK3	0.49	4.66	0	3.8	3.73
JAK3	3.23	7.89	9.35	0.54	8.54
KALRN	2.6	5.72	5.74	7.18	3.74
KDR	0.58	5.7	12.57	11.1	7.69
KIT	0	4.32	2.14	6.53	5.28
KSR1	3.65	3.43	2.98	4.12	1.78
KSR2	0.58	2.46	0	2.14	1.27
LCK	0.86	3.2	1.6	0	0
LMTK3	0.93	5.69	4.82	0	4.37
LRRK1	0	7.23	4.14	6.15	0
LRRK2	6.08	4.17	9.68	5.38	4.83
MAP3K15	2.12	6.1	5.54	6.59	5.03
MAP4K1	4.39	6.21	7.12	5.06	7.02
MAPK10	1.58	6.31	7.74	6.16	6.97
MAPK15	1.76	4.34	6.1	3.7	3.37
MAPK4	0	1.59	0.96	2.6	3.07
MARK2	6.6	6.39	6.62	5.8	6.08
MARK4	6.3	7.05	7.88	6.34	6.48
MAST1	4.85	4.45	5.46	5.49	5.03
MATK	1.26	1.8	0	2.37	0.99
MERTK	4.77	7.32	8.35	7.03	6.62
MOS	0	4.57	0.56	0	0.96
MUSK	0	2.9	0.96	0	0
MYLK3	1.26	3.83	3.13	1.58	1.95
MYLK4	5.64	3.62	6.22	4.71	4.44
MYO3A	0	0.55	6.11	0.54	1.99
MYO3B	0	0	0.99	2.89	0.96
NEK10	2.95	6.25	5.97	6.52	4.25
NEK11	4.11	5.28	8.38	7.92	7.78
NEK5	0	2.16	0.61	1.24	2.54

NIM1	0	0	3.67	6.19	4.27
NPR1	8.39	1.57	1.36	2.64	0.96
NRK	0	0.55	5.68	0.93	2.15
NTRK1	0.93	2.96	2.59	6.37	5.03
NTRK2	1.21	1.02	5.95	9.29	3.13
NTRK3	6.45	5.58	4.77	2.05	8.27
OBSCN	3.19	4.68	5.27	5.85	4.24
PAK3	0	0	1.36	6.31	3.95
PAK6	9.53	2.59	7.73	2.09	8.73
PAK7	0	2.36	0.61	4.25	0.56
PDGFRA	1.82	1.35	1.99	1.06	3.56
PHKG1	3.94	4.36	7.3	4.17	5.54
PKDCC	0	3.38	4.66	5.69	6.35
PLK5	0	0	2.47	1.81	0.59
PNCK	2.71	4.21	5.25	4.18	7.28
PRKAA2	3.51	2.88	7.94	10.68	5.44
PRKACG	0	4.07	2.19	0	0.99
PRKCB	0	0	1.03	6.79	1.99
PRKCG	0	2.33	2.56	2.03	2.3
PRKCH	6.33	6.25	5.81	7.24	8.78
PRKCQ	5.79	1.57	2.8	0	1.58
PRKD1	3.74	6.11	9.03	6.37	9.6
PRKG1	2.89	2.85	5.47	4.89	10.48
PRKG2	2.1	9.3	5.55	7.57	8.9
PSKH2	0	1.78	3.57	0	1.34
PTK6	3.92	1.02	5.85	4.2	4.02
RET	2.34	3.51	1.81	2.09	0.59
RIPK3	4.7	4.92	6.46	0	8.15
ROR2	1.49	8.26	8.63	9.15	4.43
ROS1	1.89	0	0	4.24	9.04
RPS6KA6	0	2.75	7.51	5.48	9.23
SBK1	1.86	3.78	3.75	2.03	2.79
SBK2	0.86	3.17	2.36	0	0
SGK2	4.04	6.73	6.93	3.96	4.67
SGK494	6.68	6.9	7.93	7.01	5.86
SRMS	0.86	0	0	0	0
SRPK3	0.49	3.14	5.67	2.84	6.85
STK31	1.26	3.49	2.86	3.71	1.32
STK32A	0	0.6	2.3	2.18	1.99
SYK	0	0	8.36	7.17	0.59
TIE1	0	4.1	0	0.7	0

TNK1	5.18	3.92	4.13	2.5	2.3
TRPM6	0	3.21	1.99	3.73	1.81
TSSK3	0.58	5.18	4.21	2.78	2.43
TSSK4	0	0	0	0	0
TTBK1	0	0	1.03	0	1.01
TXK	1.6	2.69	6.16	7.63	2.99
WEE2	0	0.6	2.46	0.7	1.32
WNK2	0.93	8.09	7.84	10.32	5.84
WNK3	0	4.23	3.37	8.64	6.95
ZAP70	3.58	4.29	3.43	4.1	4.34

Supplementary Table 2: RT-qPCR Primer sequences

target gene symbol	FW primer sequence	REV primer sequence
ULK4	5'-GCTCATTTACTGACATCCAAGG-3'	5'-AGGCTCTCCAAGTGCTTCTC-3'
NPR1	5'-AAGCCCTGAAGATCCACTTG-3'	5'-GTCCGAACCTTGCCTTTG-3'
CDKL4	5'-AGATGTTCATCCTGTGGCTCT-3'	5'-AAAGTAGGAGCTCTCCAGGAGTT-3'
WNK2	5'-ACCCAGAAGCTGCAAGACAT-3'	5'-GTGGCATCCCCACTCCT-3'
KDR	5'-GAACCAGAAGTAAAAGTAATCCCAGA-3'	5'-AGATAATTTGGTTCTGTCTTCCAAAG-3'
FGFR3	5'-TTTGACCGAGTCTACACTCACC-3'	5'-AGGGATGCCGGGGTA-3'
